# Endoplasmic reticulum stress—a key guardian in cancer

**DOI:** 10.1038/s41420-024-02110-3

**Published:** 2024-07-30

**Authors:** Wenlong Zhang, Yidan Shi, Linda Oyang, Shiwen Cui, Shizhen Li, Jinyun Li, Lin Liu, Yun Li, Mingjing Peng, Shiming Tan, Longzheng Xia, Jinguan Lin, Xuemeng Xu, Nayiyuan Wu, Qiu Peng, Yanyan Tang, Xia Luo, Qianjin Liao, Xianjie Jiang, Yujuan Zhou

**Affiliations:** 1 Hunan Key Laboratory of Cancer Metabolism, The Affiliated Cancer Hospital of Xiangya School of Medicine, Central South University/Hunan Cancer Hospital, Changsha, Hunan China; 2 Hengyang Medical School, University of South China, Hengyang, Hunan China; 3 The High School Attached to Hunan Normal University, Changsha, Hunan China; 4 Hunan Engineering Research Center of Tumor Organoids Technology and Application, Public Service Platform of Tumor Organoids Technology, Changsha, Hunan China; 5 Department of Oncology, Hunan Provincial People’s Hospital (The First-Affiliated Hospital of Hunan Normal University), Changsha, Hunan China

**Keywords:** Endoplasmic reticulum, Cancer microenvironment

## Abstract

Endoplasmic reticulum stress (ERS) is a cellular stress response characterized by excessive contraction of the endoplasmic reticulum (ER). It is a pathological hallmark of many diseases, such as diabetes, obesity, and neurodegenerative diseases. In the unique growth characteristic and varied microenvironment of cancer, high levels of stress are necessary to maintain the rapid proliferation and metastasis of tumor cells. This process is closely related to ERS, which enhances the ability of tumor cells to adapt to unfavorable environments and promotes the malignant progression of cancer. In this paper, we review the roles and mechanisms of ERS in tumor cell proliferation, apoptosis, metastasis, angiogenesis, drug resistance, cellular metabolism, and immune response. We found that ERS can modulate tumor progression via the unfolded protein response (UPR) signaling of IRE1, PERK, and ATF6. Targeting the ERS may be a new strategy to attenuate the protective effects of ERS on cancer. This manuscript explores the potential of ERS-targeted therapies, detailing the mechanisms through which ERS influences cancer progression and highlighting experimental and clinical evidence supporting these strategies. Through this review, we aim to deepen our understanding of the role of ER stress in cancer development and provide new insights for cancer therapy.

## Facts

- Endoplasmic reticulum stress (ERS) plays an important role in tumor development.
- The unfolded protein response (UPR) is the main response of the endoplasmic reticulum (ER) to external stimuli.
- Targeting endoplasmic reticulum stress (ERS) may be a potential strategy for cancer therapy in the future.

## Open questions

- What are the pathways by which endoplasmic reticulum stress (ERS) acts in different cancers?
- How does endoplasmic reticulum stress (ERS) affect the tumor microenvironment?
- How to improve the effectiveness of cancer treatment by combining anti-ERS with anti-tumor drugs?

## Introduction

The endoplasmic reticulum (ER) is the central biosynthetic hub of the cell. It orchestrates the synthesis of proteins (secretory and transmembrane proteins, along with certain cytoplasmic proteins) and lipids [1, 2]. According to the presence or absence of ribosomes on the surface, ER is dichotomized into the smooth ER (without ribosomes) and rough ER. The smooth ER prioritizes lipidogenesis, while the rough ER primarily facilitates protein synthesis and maturation, thereby functioning as a key manufacturing site for a variety of membrane-bound and secretory proteins. Additionally, the ER is essential not only for the synthesis and processing of these proteins but also for the synthesis and modification of proteins destined for other organelles, such as lysosomal proteins. In normative physiological paradigms, the ER quality control system stringently supervises the biogenesis and maturation of proteins to ensure proper conformational folding [3]. When proteins are not folded correctly, molecular chaperones identify these abnormalities and move them into the cytoplasm through a pathway opened by Hrd1-mediated autoubiquitination [4], subsequently subjecting them to the rigors of the endoplasmic reticulum-associated protein degradation pathway (ERAD) for degradation by the 26s proteasome [5]. Conversely, when exposed to pathological stimuli such as glycosylation inhibition, disruptions in ER Ca^2+^ homeostasis, disulfide bond disruption, intracellular hypoxia, pH fluctuations, and the accumulation of damaged DNA [6, 7], the ER’s homeostatic equilibrium is disturbed. This leads to a cascade of stress responses that ensure cellular viability in adverse conditions, exemplifying the cellular adaptive mechanisms to internal environmental changes, called endoplasmic reticulum stress (ERS).

**Fig. 1:**
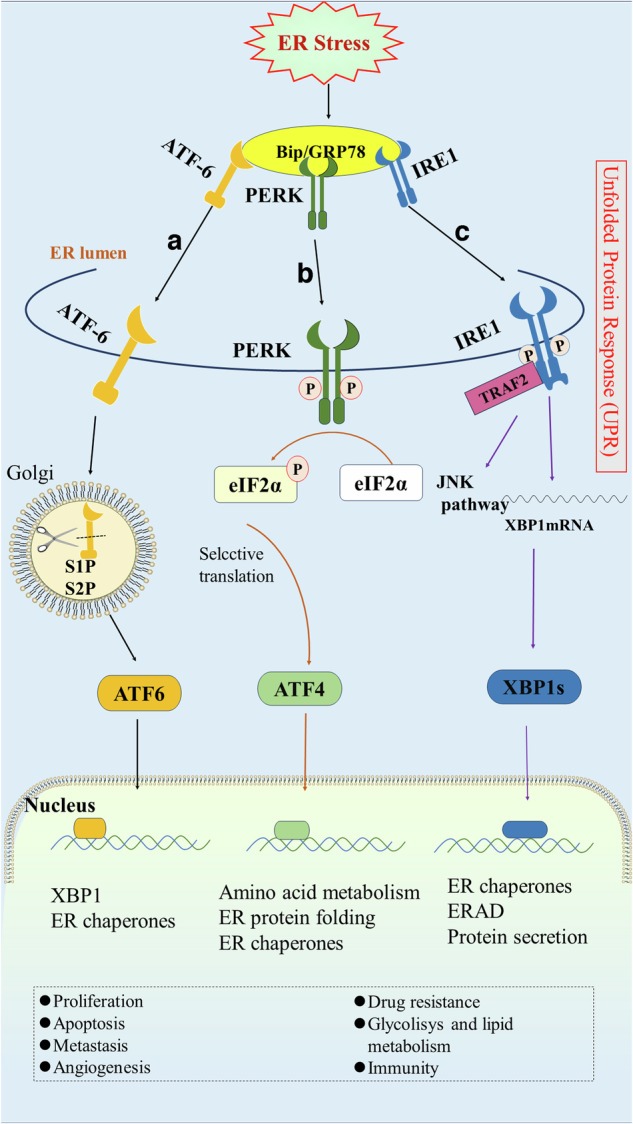
Schematic representation of the unfolded protein response in tumors. In the endoplasmic reticulum, the ERS-induced unfolded protein response (UPR) promotes tumor progression via three pathways: **a** The activating transcription factor 6 (ATF6) undergoes sequential proteolytic cleavage by site-1 and site-2 proteases (S1P and S2P), facilitating the generation of its active form. This form then translocates to the nucleus and activates numerous downstream targets. **b** Upon ERS, PKR-like ER kinase (PERK) is released from Bip/GRP78 and undergoes self-oligomerization and phosphorylation. Consequently, it phosphorylates eIF2α and activates ATF4. **c** IRE1 is released from Bip/GRP78, then undergoes dimerization and autophosphorylation, subsequently catalyzing the splicing of XBP RNA and regulating a cascade of downstream reactions.

ERS is the response of the ER to external stimuli, which may encompass the ER overload response, sterol regulatory cascade response (SRECR), and unfolded protein response (UPR) [8]. The UPR is a compensatory system triggered by disruptions caused by unfolded proteins within the ER, representing the most frequently encountered and crucial type of ERS. It entails a highly intricate mechanism, incorporating a complex network of intracellular signaling pathways. This system not only regulates the removal of misfolded proteins but is also coupled with an increase in ER size and folding capacity, thereby maintaining the homeostatic balance of the ER. The UPR process involves three ER transmembrane proteins: inositol-requiring enzyme 1 (IRE1), PKR-like endoplasmic reticulum kinase (PERK), and activating transcription factor 6 (ATF6), which function as molecular sensors that instigate the process by activating the downstream pathway; notably the Bip/GRP78 signaling cascade [9]. In the absence of unfolded proteins, these transmembrane units engage dynamically with the regulatory chaperone Bip/GRP78 within the ER. However, the accumulation of unfolded proteins induces a conformational transition in Bip, modulating its affinity for these transmembrane proteins and precipitating its release. Notably, IRE1 distinguishes itself as a principal transmembrane protein possessing dual enzymatic functionalities—kinase and ribonuclease. These are enveloped within an N-terminal luminal sensing domain and a C-terminal cytosolic effector domain [10]. Under physiological conditions, the luminal domain of IRE1α interacts with the Bip/GRP78 regulatory protein. With the onset of ERS, Bip plays a central role as a sensor, detecting subtle stress variations within the ER while simultaneously acting as an Hsp70 chaperone in its binding to IRE1α. During such stress-induced states, Bip employs its substrate-binding domain to recognize misfolded proteins, thereby triggering a decrease in the affinity of its nucleotide-binding domain for IRE1α. This sequence of molecular events culminates in the ATP-driven dissociation of IRE1α from Bip. The liberated IRE1α monomer undergoes dimerization and autophosphorylation, activating its ribonuclease function, which catalyzes the splicing of XBP1 mRNA at the proximal end of the ER membrane and encourages its transcription and translation, thus regulating a cascade of downstream reactions [11]. Analogous to IRE1, the activation of PERK is instigated by the heightened affinity of Bip/GRP78 for unfolded proteins. This facilitates the release of PERK’s luminal domain from Bip, allowing oligomerization and autophosphorylation processes to occur in PERK monomers. Upon activation, PERK phosphorylates eukaryotic initiation factor 2 (eIF2α), thereby driving the translation of downstream open reading frames—such as transcription factor ATF4—and influencing key biological procedures including amino acid biosynthesis and glycolysis [12]. ATF6 was initially recognized as an associating protein of serum response factors, existing as a type II transmembrane protein anchored to the ER [13]. This protein is characterized by its transient activity and a notably brief half-life of just 2 h, ranking it amongst the cohort of short-lived proteins [14]. In contrast to PERK and IRE1, ATF6 activation occurs within the Golgi apparatus. Here, resident ATF6 undergoes proteolytic cleavage by S1P and S2P proteases, giving rise to an active ATF6 variant that regulates several downstream elements including Bip/GRP78, GRP94, ERAD components, XBP1, the 58 kDa protein (P58IPK/DNAJC3), and CHOP, thereby playing a pivotal role in directing the UPR [15–18]. Moreover, ATF6 participates in heterodimerization with XBP1, a downstream effector of IRE1, enhancing ERAD. Importantly, this heterodimer exhibits increased affinity for UPR compared to its XBP1 homodimer counterpart under similar conditions [16] (Fig. 1).

**Fig. 2:**
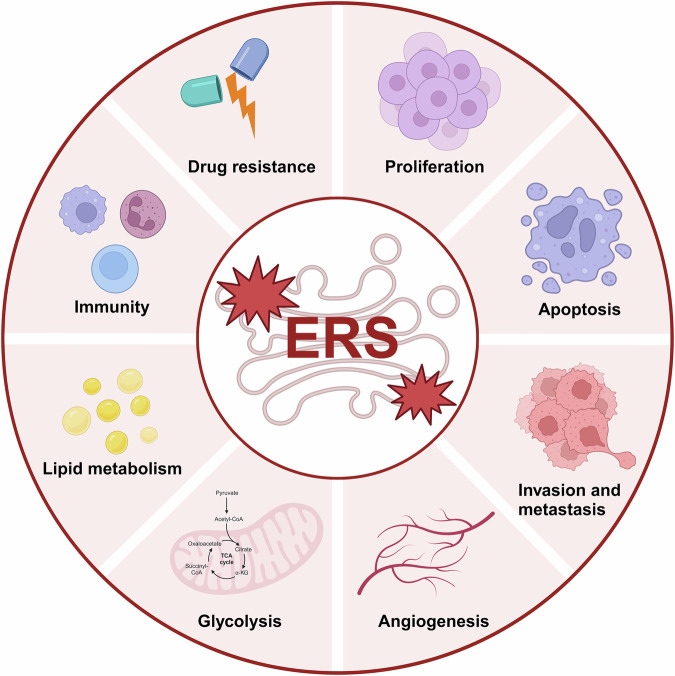
The roles of ERS in tumor progression. The endoplasmic reticulum (ER) serves several crucial functions in tumors, including the promotion or inhibition of tumor proliferation, induction of cell apoptosis, enhancement of tumor invasion and metastasis, stimulation of tumor angiogenesis, facilitation of tumor cell glycolysis and lipid metabolism, support of tumor immune evasion, and mediation of drug resistance.

ERS has been implicated as a central factor in diverse pathologies, including type II diabetes, toxoplasma infection, and ischemia/reperfusion injury [19–21]. A convergence of modern research findings underscores the crucial role of ERS within the oncogenic narrative, shaping cancer progression from inception through to advancement. This review examines the role and molecular foundations of ERS in cancer, delving specifically into its influence on various biological processes such as proliferation, apoptosis, metastasis, angiogenesis, drug resistance, cellular metabolism, and immune response (Fig. 2). The ensuing discourse will offer insights into the complexities of ERS in tumorigenesis and cancer evolution, providing a strategic blueprint for the design of cancer therapeutics.

## The role of ERS in cancer

The advancement of neoplasms is invariably influenced by a spectrum of factors intrinsic to the tumor microenvironment (TME), including hypoxia, pH fluctuations, reactive oxygen species (ROS), and a variety of cellular metabolites. In this milieu, the ER orchestrates a complex response to external stimuli, facilitating the ongoing growth of tumors. This discussion seeks to elucidate the pivotal role and intricate molecular mechanisms of the UPR within oncogenic paradigms. It will explore its ramifications on tumor proliferation, apoptosis, metastasis, angiogenesis, drug resistance, cellular metabolism, immune response, and the resilience of neoplasms to pharmacological interventions.

### Proliferation

**Fig. 3:**
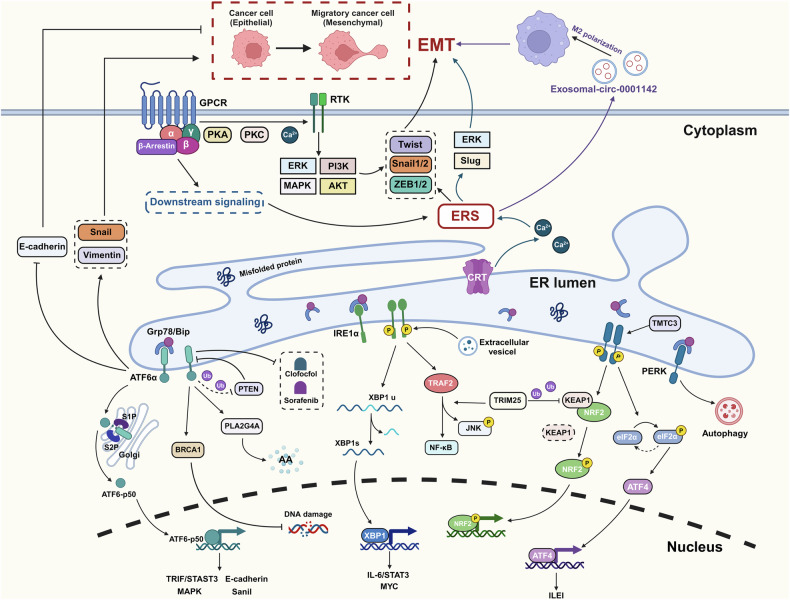
The role of ERS in tumor growth, invasion, and metastasis. Growth: ERS regulates tumor growth through signaling pathways including IRE1/XBP1/IL-6/STAT3, IRE1/TRAF2/NF-κB, PERK/NRF2, PERK/eIF2α/ATF4, and ATF6/STAT3. Metastasis: ERS regulates tumor metastasis mainly through modulation of tumor cells EMT or polarization of tumor-associated macrophages.

The unrestrained division and rapid proliferation of cancerous cells are essential for their accelerated growth. Within this dynamic, tumor cells are constantly influenced by a number of intracellular and extracellular stimuli, ERS plays a mitigating role in facilitating tumor cell proliferation [22] (Fig. 3). In hepatocellular carcinoma (HCC), TRIM25 has been found to target and degrade Keap1, consequently activating Nrf2 and fostering HCC cell proliferation by modulating the UPR signaling pathway and ERAD. Specifically, TRIM25 mediates its effects via the IRE1–JNK branch of the UPR pathway under conditions of ERS, functioning as a downstream effector [23]. In melanoma, UPR activation catalyzes tumor cell proliferation via the IL-6/STAT3 axis [24]. In non-small cell lung cancer (NSCLC), elevated expression of PRL11 promotes tumor cells proliferation through UPR and autophagy [25]. In addition, IRE1–XBP1 axis, the downstream of the UPR pathway could significantly accentuate MYC-driven tumorigenic progression in breast cancer and urothelial carcinomas [26]. Similarly, extracellular vesicles from cancer cells exploit the UPR’s IRE1α pathway to instigate oncogenic transformation in bladder cancer [27]. The pro-tumorigenic influence of IRE1α is proposed to be related to its phosphatase function, wherein in reaction to ERS stimuli, IRE1α recruits TRAF2 through its phosphatase domain, eventually prompting the activation of c-Jun N-terminal kinase (JNK) and nuclear factor κB (NF-κB) pathways and thereby bolstering oncogenesis [28, 29]. Conversely, in the context of ERS signaling, the PERK pathway exerts a tumor-suppressive effect, particularly in breast cancer, where PERK activation significantly inhibits tumor proliferation. However, the molecular intricacies of this phenomenon remain to be elucidated [30].

ATF6 is emerging as a key downstream effector in the field of ERS. It has a spectrum of roles in oncological biology. It has been elucidated that elevated ATF6α is closely associated with the progression of prostate cancer. Furthermore, it could regulate arachidonic acid metabolism via the ATF6α–PLA2G4A signaling pathway to promote prostate cancer progression [31], or promote the malignancy through a reciprocal negative feedback mechanism with PTEN [32]. In addition, ATF6 plays a pivotal role in maintaining BRCA-1 expression. This enables colon cancer cells to evade DNA damage and enhance their viability [33]. In colorectal cancer, phosphorylated ATF6 has been demonstrated to promote tumor development by inducing gut microbiome dysbiosis and activating the TIR domain-containing adapter-inducing interferon-β (TRIF)/STAT3 signaling cascade [34]. Moreover, ATF6 has been demonstrated to accelerate the proliferation of cervical cancer cells via the MAPK pathway [35]. However, ATF6 exhibits a tumor suppressor effect when combined treatment with clofoctol and sorafenib in prostate cancer [36].

In certain circumstances, the inhibition of ERS can facilitate tumor survival. For example, in BRAF-mutant uveal melanoma, inhibiting ERS often promotes the survival of UM cells [37]. This inhibitory effect may be related to ERS-associated autophagy. For instance, in breast cancer, PRL5 downregulates E2F1 expression, which in turn enhances the transcriptional activation of Bip/GRP78, suppresses the ERS response, and promotes autophagy [38]. Therefore, ERS-mediated autophagy is likely crucial for cell survival [39]. The primary objectives of autophagy are to rectify energy imbalances, ensure proper protein folding, and recycle cellular contents [40]. Generally, under ERS, the UPR detects the accumulation of misfolded proteins, leading to the activation of ATG genes (autophagy regulators) [41]. It is noteworthy that during this process, IRE1 and PERK are more significantly involved in stimulating autophagy [42]. In pancreatic cancer cells (PANC-1), fisetin induces protective autophagy by regulating the levels of IRE1, PERK, and ATF6 through the p8-p53/PKC-α pathway. Autophagy inhibitors CQ and 3-MA can enhance the anti-tumor effects of fisetin [43]. In gliomas, flavokawain B induces autophagy in GBM cells through the ERS-dependent ATF4–DDIT3–TRIB3–AKT–MTOR–RPS6KB1 signaling pathway to inhibit proliferation [44]. Similarly, combined treatment with autophagy inhibitors CQ proves to be an effective anti-tumor strategy in pancreatic cancer cells. ERS-related autophagy is also observed in lung adenocarcinoma, where interferon gamma (IFN-γ) induces ERS and UPR in lung adenocarcinoma cells by activating the JAK1/2–STAT1 and AKT–mTOR signaling pathways. IFN-γ-induced UPR subsequently reduces the expression of LAMP-1 and LAMP-2, impairing autophagic flux [45]. Additionally, DEH inhibits the growth of colorectal cancer cells by activating the PERK/eIF2α and IRE1α/XBP1s/CHOP pathways, thereby stimulating autophagy [46].

These findings highlight the role of ERS during cancer progression. The UPR’s downstream branches exhibit divergent impacts across various malignancies, either as oncogenic drivers or suppressors, which were influenced by the intrinsic characteristics of the tumor and the interplay between the different branches of the signaling pathway. In addition, the functionality of ATF6 is complex and its paradoxical effects in prostate cancer may be related to the pharmacological agents used. Further investigation is needed to unravel the complex underlying mechanisms.

### Apoptosis

Apoptosis operates as an inherent mechanism, aiding organisms in sustaining homeostasis within their internal environment. Conventionally, ERS serves a beneficial function, managing the synthesis and processing of a variety of proteins, including secreted and membrane-bound proteins. Nonetheless, during instances of prolonged or intense ERS, tumor cells inevitably encounter failure in re-establishing normal ER functions. This results in cellular dysfunction, ultimately triggering cell death.

**Fig. 4:**
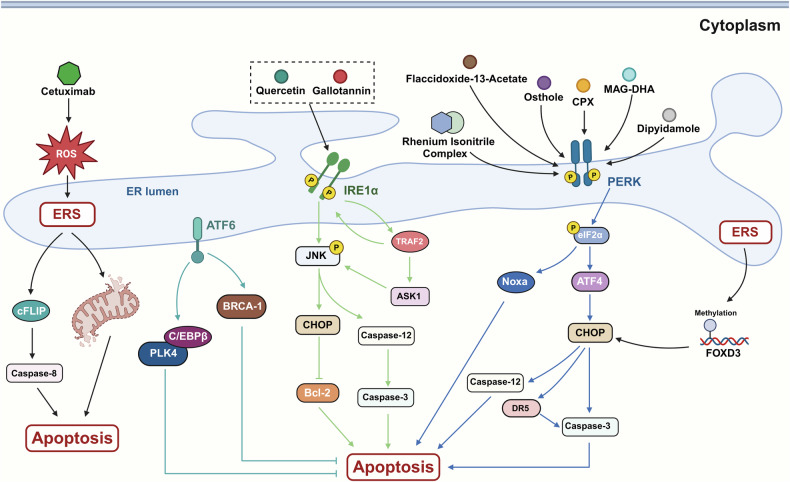
Effect of endoplasmic reticulum stress on tumor cells apoptosis. IRE1 induce tumor cells apoptosis via IER1/TRAF2/caspase-12, IRE1/ASK1/JNK/BCL-2, and IRE1/JNK/BCL-2 signaling; PERK regulated tumor cells apoptosis via PERK/eIF2α/ATF4/CHOP/DR5/caspase-3 (caspase-12) signaling; ATF modulated apoptosis via ATF6/CHOP/DR5/caspase-3 (caspase-12) signaling.

It has been demonstrated that ERS can induce tumor cell apoptosis through the IRE1/TRAF2/ASK1/JNK pathway, the caspase-12 kinase pathway, and the C/EBP homologous protein (CHOP)/GADD153 pathway [47–52] (Fig. 4). Specifically, the caspase-12 pathway and the CHOP/GADD153 pathway may be delineated as IRE1/TRAF2/caspase-12 and PERK/ATF4/CHOP, respectively. These apoptotic signaling pathways may induce apoptosis by promoting the upregulation of downstream apoptotic effector protein caspase-3 [47, 49–52]. Additionally, ERS can suppress Bcl-2 expression via the IRE1–JNK–CHOP pathway, thereby inducing apoptosis in gastric cancer cells [53, 54]. The IRE1/TRAF2/ASK1/JNK cascade is initiated when IRE1 recruits TRAF2 to form the IRE1–TRAF2 complex. This subsequently activates apoptosis signal-regulating kinase 1 (ASK1), which in turn activates JNK, ultimately leading to apoptosis [47, 48]. The CHOP, a specific protein of ERS, is intrinsic to ERS and a member of the CCAAT/enhancer-binding proteins (C/EBPs) family. It is usually expressed at a minimal level. In the context of ERS, transcription factors such as ATF4, ATF6, and XBP1 translocate into the nucleus, where they enhance CHOP transcription. This ultimately leads to the activation of the apoptotic effector caspase-3, thus triggering tumor cell apoptosis [55]. Previous studies have demonstrated that quercetin can induce apoptosis in cervical cancer cells by activating the ERS response, although the precise mechanisms underlying this process remain unclear. Experimental data indicate that the levels of Bax, Bcl-2, GRP78, CHOP, and IRE1 increase in correlation with the concentration of quercetin [55]. This inference implies a potential linkage between apoptosis in cervical cancer cells and the IRE1–JNK–CHOP pathway, presenting a prospective novel therapeutic strategy for cervical cancer. In addition, shikonin may stimulate the death of gastric cancer cells via this pathway. Such an observation accentuates the importance of the IRE1/JNK/CHOP signaling axis in modulating tumor cell apoptosis [54].

In hypoxic conditions, osthole initiates the PERK/eIF2α/ATF4/CHOP/DR5 signaling cascade within the UPR, thereby upregulating the expression of caspase-3 and promoting apoptosis in colon cancer cells [56]. Similarly, docosahexaenoic acid monoglyceride (MAG-DHA) activates the PERK/eIF2α/CHOP pathway and upregulates the expression of the lytic enzyme caspase-12, thereby promoting apoptosis of breast cancer cells [57]. The PERK/eIF2α/ATF4 pathway also plays a role in cyclophosphamide-induced apoptosis in colorectal cancer cells. Furthermore, it has been demonstrated to promote apoptosis in chemoresistant (Hct-8/5-FU) and chemosensitive (Hct and DLD-1) colorectal cancer cells [58]. The methylation of FOXD3 under conditions of ERS results in the augmented expression of proteins associated with the UPR, followed by the upregulation of the CHOP protein, which consequently promotes apoptotic processes [59]. It is noteworthy that the downregulation of cFLIP represents a crucial early step in the ERS-induced apoptosis pathway in colorectal cancer cells [60]. In bladder cancer, flacidoxide-13-acetate induces apoptosis by activating the PERK/eIF2α/ATF6/CHOP signaling axis. Gamma-tocotrienol, on the other hand, promotes apoptosis by activating the PERK/eIF2α/ATF4 signaling pathway via ERS [61], it is reasonable to posit that apoptosis in bladder cancer cells may also be mediated through this pathway. Additionally, ERS-induced apoptosis has been reported in glioma cells [62, 63]. In glioma cell models U87 and T98G, dipyridamole induces apoptosis by regulating the PERK/eIF2α signal pathway to activate the BH3-only protein Noxa [51]. The PERK pathway-mediated apoptosis generally follows the PERK/eIF2α/ATF4/CHOP pathway, similarly demonstrated by [Re(CO)_3_(dmphen)(*p*-tol-ICN)] + (TRIP)-mediated cancer cell death [64]. Additionally, research on cetuximab-induced apoptosis in laryngeal squamous cancer cells indicates that ROS play a significant role in ERS-mediated apoptosis [65]. The findings provide insights for future investigations into ER-mediated tumor cell apoptosis. ER-induced apoptosis of tumor cells is also notably observed in neuroblastoma. Earlier studies with Hela cells demonstrated that fenretinide (4HPR) induces tumor cell death and procaspase activation via the PERK/eIF2α signaling pathway [66]. Further research indicated that in neuroblastoma, fenretinide achieves its anti-tumor effects by inducing ERS, specifically through upregulating ATF4 transcription levels, thereby promoting apoptosis [67]. Protein disulfide isomerase (PDI) is a foldase and molecular chaperone essential for the formation, breakage, and rearrangement of disulfide bonds in unfolded or misfolded proteins, playing a critical role in maintaining ER protein homeostasis. Dysregulation of PDI can impair the protein-folding efficiency within the ER lumen, resulting in the accumulation of unfolded and misfolded proteins, which in turn triggers ERS [68]. Evidence has shown that PDI is overexpressed in breast cancer [69, 70]. DDA, a PDI inhibitor, has been demonstrated to induce breast cancer cell death in both in vitro (MDA-MB-468) and in vivo (BT474 mouse xenograft model) studies. The underlying mechanism may involve DDA targeting PDI family members, such as PDIA1, AGR2, and ERp44, thereby initiating DR4 and DR5-mediated caspase 8 and 3 activation, leading to apoptosis [71].

As a general rule, ERS can instigate apoptosis through several downstream signaling pathways. The most commonly implicated pathways involve IRE1 and PERK, whereas ATF6, frequently observed serving an anti-apoptotic role, is less prevalent. A multitude of studies have suggested that upon activation in osteosarcoma cells, ATF6 interacts with the polo-like kinase 4 promoter to enlist C/EBPβ, subsequently inhibiting apoptosis in these cells [72]. In colon cancer cells, it conserves the expression of BRCA-1, thereby shielding the cells from cytotoxic effects mediated by ER stressors such as DPE and thapsigargin (TG) [33].

### Invasion and metastasis

The intricacies of tumor invasion and metastasis represent a complex phenomenon, with the underpinning mechanisms still largely elusive. Research has underscored the substantial influence of ERS not only on modulating tumor growth and apoptosis but also on governing tumor invasion and metastasis.

The invasion and metastasis of tumors mediated by ERS are influenced by multiple factors (Fig. 3). Initially, the process of invasion and metastasis of tumors is linked to ERS-regulated epithelial–mesenchymal transition (EMT), a transformation characterized by the reduction of epithelial markers and an increase in mesenchymal markers, which significantly contributes to the invasiveness of tumor cells [73]. Within ERS, the UPR collaboratively regulates EMT with G-protein coupled receptors (GPCRs), impacting tumor invasion and metastasis. GPCRs, upon binding to ligands with trimeric proteins and β-arrestin on the cellular membrane, initiate a cascade involving downstream UPR signals such as IRE1, PERK, and ATF6. Concurrently, through interactions involving PKA, PKC, calcium ions, and receptor tyrosine kinases, GPCRs facilitate the activation of pathways like ERK/MAPK and PI3K/AKT. These pathways, in concert with active ERS, upregulate the expression of EMT transcription factors (Snail1/2, Twist, and ZEB1/2), further activating EMT [74]. For instance, in a cervical cancer model, the ATF6 branch of UPR can promote EMT and further tumor invasion and metastasis by reducing the expression of E-cadherin and upregulating the expression of the major transcription proteins Snail and vimentin through the MARK signaling pathway [35]. In addition, calreticulin facilitates both acute and chronic free calcium-dependent ERS by activating the Slug and ERK signaling pathways and subsequently promoting EMT [75]. And ERS may enhance tumor invasiveness and metastasis by promoting the release of exosomes, which are specialized extracellular vesicles encapsulating diverse RNA molecules and proteins, generally 50–200 nm in diameter [76]. Research indicates that the CXCL12/CXCR4 axis is closely related to liver metastasis of colorectal cancer, and exosome-encapsulated miRNAs can promote this process by enhancing the polarization of M2 macrophages [77]. In breast cancer, ERS facilitates metastasis by inducing the release of exosome circ_0001142, which polarizes M2 macrophages [78].

In summary, ERS generally facilitates the migration of the majority of tumor cells. However, in certain tumors, such as epithelial ovarian cancer, it paradoxically exerts an inhibitory influence. The exact mechanisms behind this effect remain elusive, potentially tied to the distinct characteristics inherent to various tumor types.

### Angiogenesis

**Fig. 5:**
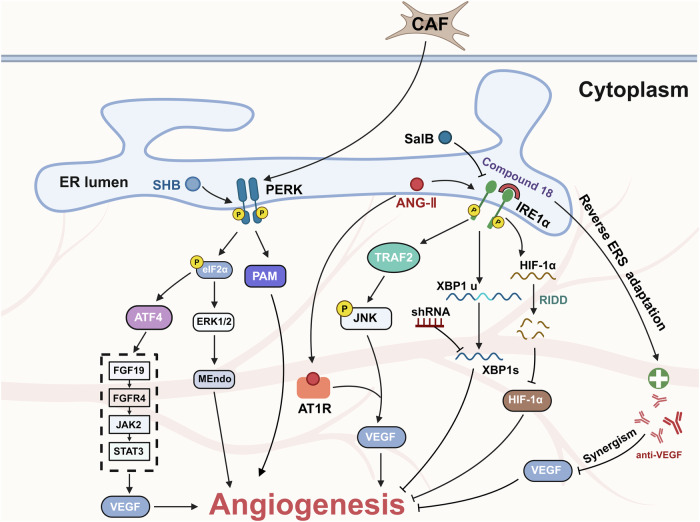
The role of ERS in tumor angiogenesis. Endoplasmic reticulum stress (ERS) plays a pivotal role in the modulation of tumor angiogenesis via a myriad of interwoven pathways, encompassing IRE1α, PERK, BIP, ATF6, and the secretion of exosomes. Within the IRE1α signaling axis, certain molecules, such as SaIB and ANG-II, have been implicated in either activating or repressing the angiogenic process. Along the PERK pathway, entities like SHB and cancer-associated fibroblasts (CAFs) facilitate angiogenesis through the manipulation of the PERK cascade. In contrast to these mechanisms, a reduction in BIP expression or the presence of tumor-derived exosomes tends to exert an inhibitory influence on angiogenesis regulation.

Solid tumors are characterized by a microenvironment deficient in oxygen and nutrients due to the significant consumption necessitated by the rapid proliferation and invasive metastasis of tumor cells. This deficiency can be replenished via tumor angiogenesis. Under hypoxic conditions, ERS facilitates angiogenesis through the downstream pathway of UPR (Fig. 5). However, ERS does not universally promote angiogenesis. In certain contexts, it might suppress tumor vascular development, as exemplified when angiogenesis is inhibited by tumor-secreted exosomes induced by ERS [79, 80].

Hypoxia-inducible factor-1 (HIF-1), a key protein within the TME, is known for its role in promoting the transcription of molecules such as the vascular endothelial growth factor (VEGF) [81]. Studies have suggested that XBP1, a downstream molecule of the IRE1 pathway, can facilitate tumor angiogenesis independently of VEGF [82]. In model systems employing human umbilical vein endothelial cells, IRE1 knockdown significantly impairs HIF-1α protein expression under hypoxic conditions. This inhibition appears to primarily depend on the endonuclease activity of IRE1, rather than its downstream effector XBP1s [83]. Notably, in gliomas, IRE1 inhibition results in marked downregulation of angiogenesis-related molecules, principally those that are downstream targets of HIF-1. This could be attributed to IRE1’s role in stabilizing HIF-1 protein [84]. Moreover, inhibiting IRE1α has been observed to reverse ERS adaptation, thereby enhancing the efficacy of anti-angiogenic therapies in triple-negative breast cancer [85]. The IRE1 pathway of the UPR collaborates with ANG-II to foster tumor angiogenesis. Specifically, Ang-II triggers the IRE1/JNK/P38 signaling cascade of ERS and concomitantly upregulates VEGF expression through interactions with its AT1R receptor [86, 87]. Recent findings show that salubrinal acid B, a novel type I IRE1 kinase inhibitor, suppresses angiogenesis by downregulating this pathway [88]. These insights underscore multiple mechanisms through which IRE1 fosters tumor angiogenesis.

In addition to IRE1 downstream factors XBP1 and HIF-1α, PERK downstream factors such as ATF4 and ATF6 have been shown to interact with vascular endothelial factor promoters, thereby augmenting their expression [89–91]. PERK has been implicated in promoting the expression of a plethora of angiogenic factors [92]. In gliomas, it has been demonstrated that the expression of peptidylglycine alpha-amidating monooxygenase mediated by PERK stimulates angiogenesis, thereby accelerating tumor growth [93]. Moreover, in pancreatic ductal adenocarcinoma (PDAC), cancer-associated fibroblasts (CAFs) can contribute to tumor microvasculature formation and subsequent tumor progression by serving as sources of endothelial cells. This is largely facilitated through the activation of the PERK–eIF2α–ERK1/2 signaling pathway in CAFs, driving the transition from mesenchymal to endothelial phenotypes and thereby promoting tumor vascularization [94]. Among the three primary downstream branches of the UPR, ATF6’s role in fostering angiogenesis within tumors has been relatively underexplored.

Hence, ERS can either promote or hinder tumor angiogenesis, setting the stage for inventive therapeutic strategies that exploit ERS inhibition to boost anti-angiogenic responses. Furthermore, beyond the direct effects of ERS, tumor exosomes associated with this stress phenomenon can modulate angiogenesis, predominantly by inhibiting it.

### Drug resistance

Chemotherapy has long served as a cornerstone treatment for various cancers, yet its effectiveness can sometimes be suboptimal among certain patients. In addition to the notable side effects associated with chemotherapy, sustained treatment often leads to the development of drug resistance within tumor cells. Comparable challenges have emerged with recent advances in immunotherapies and targeted therapies. Consequently, a pivotal focus in clinical oncology research involves augmenting tumor cell susceptibility to chemotherapy and other therapeutic agents. Past investigations have delineated five mechanisms through which tumors may develop resistance: (1) reduced drug uptake; (2) genetic determinants (including gene mutations, amplifications, and epigenetic alterations associated with microRNAs); (3) augmented autocrine production of growth factors; (4) repair of drug-induced DNA damage; and (5) heightened metabolism of xenobiotics [95].

First, tumor drug resistance associated with ERS correlates with downstream pathways of the UPR. A previous study showed that ATF6 mediates chemotherapy resistance in cancer cells by promoting their survival [96]. Recent analyses of ERS and resistance in HCC have shown that ATF6 activated P58 via its protease activity [97]. For example, colorectal cancer patients initially positive for EGFR exhibited significant sensitivity to cetuximab, yet ultimately developed resistance [98]. Carfilzomib, targeting K-RAS(+) and cetuximab-resistant colorectal cancer, operates by upregulating UPR downstream CHOP and ATF6 expression, and enhancing apoptotic pathways through caspase-3/7 for anti-tumor activity [99]. It has been demonstrated that ATF6 sustains activation of the mammalian target of rapamycin (mTOR) pathway, a vital element in tumor metabolism [100]. Further research underscores the crucial role of ATF6 in DNA repair, which is achieved by maintaining BRCA-1 expression in colon cancer cells under ERS. This subsequently elevates sensitiveness to doxorubicin by intensifying the cytotoxic effects of ERS-inducing drugs [33]. This underscores the potential of ATF6-targeted therapies as novel chemosensitizers in colorectal cancer treatment. Earlier research on the PERK pathway revealed that PERK induces tumor drug resistance by upregulating ABC transporters or activating Nrf2 to stimulate autophagy [96]. For example, in chemoresistant human colorectal adenocarcinoma HT29 cells, both ABCC1 and Nrf2 are upregulated [101]. Present studies indicate that PERK primarily enables drug resistance through the PERK–eIF2α–ATF4–CHOP pathway by activating autophagy [97]. In the IRE1 pathway, phosphorylated IRE1 upregulates ABC transporter expression, inhibiting cell cycle arrest and apoptosis, thereby fostering drug resistance, or by altering drug transport via splicing XBP1 and activating Nrf2 [96]. This is specifically exemplified in colorectal cancer, where 5-FU activation of the XRE1–ABCB1–XBP1 pathway enhances ABC protein expression, promoting resistance to fluorouracil [102]. And new resistance mechanisms involving IRE1, such as the IRE1–TRAF2–JNK pathway enhancing DNA repair and inhibiting apoptosis, as well as affecting drug transport by splicing XBP1 and activating Nrf2, have been proposed [97]. In certain cancer types, drug resistance is orchestrated through the modulation of the IRE1 and PERK signaling pathways. Notably, triple-negative breast cancer exhibits resistance to doxorubicin, and osteosarcoma demonstrates resistance to cisplatin, among others [103, 104]. In melanoma, activating mutations in BRAF induce persistent ERS [105]. The resistance to most anti-cancer therapies is attributed to the adaptation to prolonged low-intensity ERS. For instance, this resistance is mediated by the activation of NFκB and the inhibition of apoptosis proteins via the IRE1α/TRAF2 pathway [106]. Another instance of resistance in melanoma involves vemurafenib, which induces miR-410-3p in melanoma cells through ERS. This induction promotes the transition of cells to a more invasive phenotype, resulting in resistance to BRAF inhibitors [107].

Furthermore, the resistance or sensitivity of tumor cells to therapeutic agents may be associated with BiP/GRP78, a chaperone protein that binds to monomers downstream of the UPR and stabilizes IRE1, PERK, and ATF6. It has been extensively documented that overexpression of GRP78 contributes to resistance in various cancers, such as sorafenib resistance in liver cancer [108], sunitinib resistance in renal cancer [109], and gefitinib resistance in NSCLC [110]. In pancreatic cancer models, GRP78-mediated chemotherapy resistance arises from its interaction with the extracellular domain of CLPTM1L/CRR9 on tumor cell surfaces. This interaction can be targeted by monoclonal antibodies against CLPTM1L/CRR9, enhancing chemotherapy sensitivity and unveiling a novel therapeutic approach [111]. BiP/GRP78 can also regulate nasopharyngeal carcinoma resistance to cisplatin through the modulation of exosome ERp44 [112]. Additionally, a distinctive aspect observed in cervical cancer models is that overexpression of GRP78 in cervical cancer (CVC) exhibits dual influences on cisplatin responsiveness, affecting both resistance and sensitization [113, 114]. These examples underscore the pivotal role of BiP/GRP78 in conferring resistance to chemotherapeutic and immune-targeted drugs, with targeting BiP/GRP78 emerging as a promising strategy for enhancing cancer treatment. Of course, there are many more mechanisms of tumor cell resistance mediated by ERS, including the role of hydroxymethylglutaryl-CoA synthase 1 in promoting resistance in acute myeloid leukemia through the ER–UPR–mitochondrial axis [115]. ERS-induced downregulation of PHLPP leads to chemotherapy resistance in colon cancer [116], and ERS-induced upregulation of exosome miR-301a-3p causes resistance in HER2-positive gastric cancer cells to trastuzumab [117].

Moreover, ERS does not invariably enhance tumor resistance to therapeutic agents; it can also substantially boost sensitivity by aiding some tumors in overcoming resistance. Part of this mechanism is closely related to ERS-induced apoptosis of tumor cells. For example, recent research indicates that exosome miR-512-3p has facilitated retinoblastoma cells in overcoming cisplatin resistance by promoting apoptosis triggered by ERS [118]. Numerous instances exist, such as the inhibition of phosphoglucomutase 3 (PGM3) activating UPR to counteract gemcitabine resistance in pancreatic cancer [119], and the activation of the IRE1–XBP1 pathway of UPR overcoming ibrutinib resistance in diffuse large B-cell lymphoma [120] (Table 1).

**Table 1:** Representative drugs that induce tumor sensitivity or resistance via ERS.

Pharmaceutical	Targets	Mechanism	Tumor	References
Carfilzomib	ATF6	CHOP, ATF6 caspase-3/7↑	Colorectal cancer	[99]
5-FU	IRE1	IRE1–ABCB1–XBP1	Colorectal cancer	[102]
5-FU	PERK	ABCC1↑, Nrf2↑	Colorectal cancer	[101]
Doxorubicin	ATF6	Cytotoxic effect	Colorectal cancer	[33]
Doxorubicin	IRE1, PERK	PDCD4–eIF4A–FAK pathway	Triple-negative breast cancer	[103]
Sorafenib, sunitinib, gefitinib	GRP78/BIP	Unclear	Hepatocellular carcinoma Renal cell carcinoma Non-small cell lung cancer	[108–110]
Gemcitabine	GRP78/BIP	CLPTM1L/CRR9	Pancreatic cancer	[110]
Ibrutinib	IRE1	IRE1/XBP1	Diffuse large B-cell lymphoma	[120]
Trastuzumab	Unclear	miR-301a-3p	HER2-positive gastric cancer	[117]
Cisplatin Cisplatin	Unclear IRE1,PERK	miR-512-3P NF-κB	Retinoblastoma Osteosarcoma	[118] [104]
Cisplatin	GRP78/BIP	Exosomal ERp44	Nasopharyngeal carcinoma	[112]
Cisplatin	GRP78/BIP	ATM pathway and calcium efflux	Ovarian cancer	[113, 114]
Gemcitabine	Unclear	PGM3↓	Pancreatic cancer	[119]
Imiquimod	Unclear	miR-410-3p	Melanoma	[107]
Tamoxifen	Bip/GRP78	Unclear	Breast cancer	[170]

Taken together, ERS plays an extremely important role in various cancer drug therapies, and the mechanisms by which different UPR signals affect tumor drug resistance are not the same. Notably, even a singular pathway can have profoundly disparate impacts on either enhancing sensitivity or fostering resistance to drugs in tumor cells. Thus, further exploration into the role of ERS in modulating tumor resistance could unveil novel strategies for overcoming drug resistance in cancer.

### Glycolysis and lipid metabolism

**Fig. 6:**
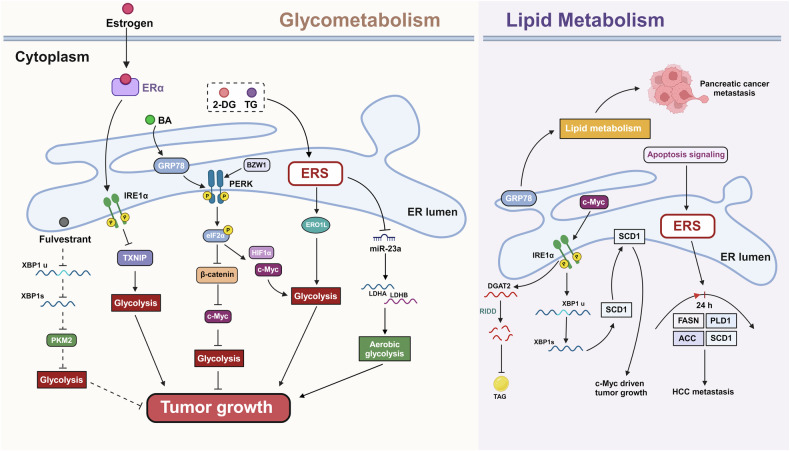
Effects of endoplasmic reticulum stress on glycolysis and lipid metabolism. Glycolysis: in tumor cells, glycolysis may be modulated by ERS-induced UPR, miRNA, or mitochondrial dysfunction. Specifically, the upregulation of BZW1 and overexpression of ERO1L promote pancreatic ductal cancer cell glycolysis through PERK. BA induces overexpression of GRP78 and activates the PERK pathway, subsequently downregulating β-catenin expression. This series of events leads to c-Myc-mediated glycolytic inhibition, thereby suppressing breast cancer cell proliferation. Estrogen enhances glycolysis and orchestrates the proliferation of breast cancer cells by decreasing the expression of TXNIP via activation of the IRE1/XBP1 pathway. The anti-proliferative effect of fulvestrant on prolactinoma GH3 cells is achieved by activating IRE1, reducing PKM2 expression, and inhibiting glycolysis. Lipid metabolism: ERS typically affects lipid metabolism through IRE1, PERK, and GRP78/BIP. IRE1 can augment SCD1 expression or mediate RIDD to reduce DGAT2 mRNA expression, thereby impacting lipid metabolism. The PERK pathway can influence lipid metabolism by controlling the expression of lipid regulatory enzymes such as FASN, ACL, and SCD1. Conversely, GRP78/BIP regulates lipid biosynthesis by modulating SREBP.

Normal cellular metabolism plays a pivotal role in maintaining physiological functions. In cancer cells, due to the demands of rapid growth and invasive metastasis, metabolic activities are abnormally amplified to support their proliferative needs. Concurrently, the accumulation of metabolic waste can negatively impact the growth of these cells. As a result, ERS aids cancer cells in adapting to various hostile environments, thus promoting their survival (Fig. 6).

Initially, ERS modulates glycolysis in a variety of cancer cells to control tumor growth. For example, in PDAC, BZW1 is overexpressed and functions as an adapter of PERK, facilitated eIF2α phosphorylation. This activation promoted internal ribosome entry site-dependent translation of HIF-1α and c-Myc, stimulating the Warburg effect and accelerating PDAC cell proliferation [121]. In addition, ERS-dependent upregulation of ERO1L also facilitates the Warburg effect, thereby promoting PDAC growth [122]. Treatment of HeLa cells with the glucose analog 2-deoxyglucose or TG induces ERS, promotes LDHA and LDHB expression by inhibiting miR-23a, thus adapting the cells to aerobic glycolysis and enhancing tumor proliferation [123]. In addition, the ERS receptor Bip/GRP78 plays a critical role in pancreatic cancer by modulating the oxidative state of cells to maintain tumor stem cell stability [124]. In highly invasive breast cancer, betulinic acid induces Bip/GRP78 overexpression and activates PERK, leading to eIF2α phosphorylation, which inhibits β-catenin expression and subsequently represses c-Myc-driven glycolysis [125]. And estrogen enhances the Warburg effect in breast cancer by activating the IRE1 signaling pathway of the UPR to suppress TXNIP expression, elucidating the pathway by which the estrogen/ERα–IRE1–TXNIP axis stimulates tumor cell growth and proliferation [126]. Low expression of XBP1, including XBP1s and XBP1u (downstream targets of IRE1), can inhibit the Warburg effect in prolactinoma GH3 cells by downregulating PKM2, and fulvestrant exerts its tumor-suppressive effects through this pathway [127].

Second, in addition to glycolysis, alterations in lipid metabolism are a major hallmark of cancer [128]. In tumor cells, ERS affects not only glycolysis but also lipid metabolism. It is well documented that lipid metabolism in tumor cells is closely linked to the IRE1 signaling pathway. For example, in tumors transformed by c-Myc, IRE1α/XBP1s stimulates tumor growth by upregulating the expression of the lipid desaturase SCD1, a key enzyme in lipid metabolism and tumor invasion located in the ER [129, 130]. In addition, studies suggested that IRE1α, through its role in regulated IRE1-dependent decay (RIDD), acts as a transcriptional repressor of DGAT2 expression in cells such as MDA-MB-231, HCC1806, and BT-549, resulting in reduced DGAT2 mRNA levels and subsequently lower triacylglycerol levels [131]. Apart from the IRE1 pathway, tumor lipid metabolism is intricately linked to the PERK pathway. Prior research has demonstrated that dysfunction in the PERK pathway leads to reduced expression of fatty acid synthesis enzymes like FASN, ACL, and SCD1, highlighting the link between PERK and the regulation of lipid metabolism [132]. In studies using the HepG-2 liver cancer model, apoptin-mediated ERS was found to drive changes in lipid metabolism, increasing the expression of lipid synthesis-related enzymes such as FASN, ACC, PLD1, and SCD1 within the first 24 h, followed by a decline [133].

This emphasizes the influence of ERS on lipid metabolism, suggesting that sustained stress may result in a diminution of lipase levels due to ER injury, consequent enzyme downregulation. Moreover, apoptin has been demonstrated to inhibit the invasion and metastasis of HepG-2 liver cancer cells, thereby presenting a new avenue for research. In summary, ERS imposes diverse effects on glycolysis and lipid metabolism within tumor cells, subsequently modulating their growth as well as the processes of invasion and metastasis.

### Immunity response

**Fig. 7:**
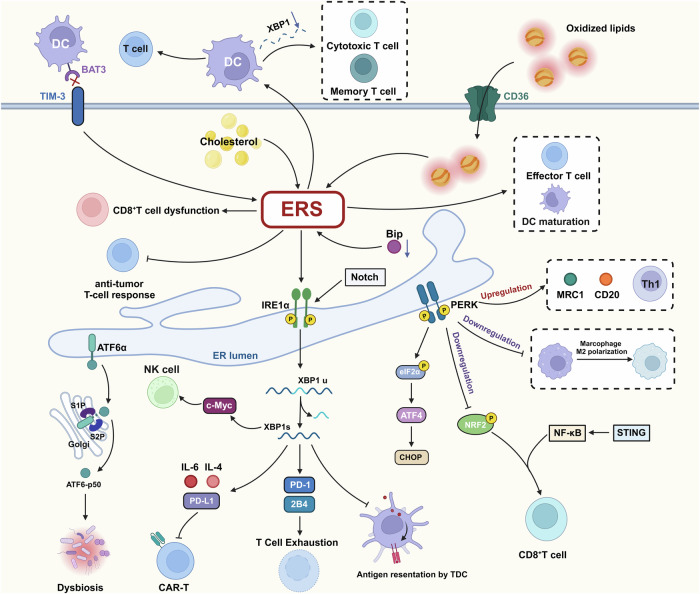
The role of ERS in tumor immunity. In tumor-associated immunity, the IRE1 arm not only suppresses T cell activity and proliferation, but also inhibits the antigen presentation capacity of dendritic cells (DC) and the proliferation of natural killer (NK) cells. The PERK arm, through its signaling pathway, can influence the expression of macrophages, DC, and T helper (Th) cells, and also plays an immunosuppressive role in myeloid-derived suppressor cells (MDSCs). Furthermore, ATF6 primarily mediates the occurrence of innate immunity in the intestine.

In certain adverse tumor environments, including nutrient deprivation, hypoxia, accumulation of inhibitory metabolites, and abnormal production of ROS, the ER balance within tumor-associated immune cell subsets is disrupted, leading to compromised anti-tumor immune responses [134] (Fig. 7). ERS induced by the TME is a major factor in the dysfunction of tumor-infiltrating T cells [135, 136], which was mainly achieved through the activation of IRE1, PERK, and CHOP driven by the TME [137–139]. It was shown that accumulation of cholesterol by CD8+ T cells in the tumor-induced expression of the inhibitory receptors PD-1 and 2B4 and promoted T cell exhaustion in an XBP1-dependent manner. Conversely, XBP1 deficiency enhanced T cell anti-tumor function and prolonged patient survival [139, 140]. And CD36-mediated uptake and oxidation of low-density lipoprotein progressively induces severe ERS in T cells, which is a major cause of CD8+ T cell dysfunction [141, 142]. In certain cancers, such as uveal melanoma, CD8+ T cell infiltration levels correlate strongly with a higher risk of disease. Reports indicate that high-risk uveal melanomas show increased infiltration of CD8+ T cells, follicular helper T cells, gamma and delta T cells, and activated NK cells, while low-risk cases show a greater presence of memory resting CD4+ T cells, naive B cells, activated and resting mast cells, monocytes, and resting NK cells [143]. The mechanism may be that in CD8+ T cells, ERS and the UPR act as extracellular immune regulators, modulating dendritic cells (DCs) through polarization types and pro-inflammatory responses, thereby promoting the production of under-proliferating T cells [144]. A study had elucidated a potential mechanism by which DCs deficient in the adapter protein BAT3, which interacts with TIM-3 on T cells, exhibit a hyperactive UPR, leading to the development of a tolerogenic phenotype that subsequently attenuates the efficacy of anti-tumor T cell responses [145]. Furthermore, tumor DCs with inactivated XBP1 can enhance the functionality of lymphocytes, such as the maturation of cytotoxic T lymphocytes and memory T lymphocytes [146]. Additional research on diffuse large B-cell lymphoma demonstrates that in myeloid macrophages, activation of the Notch-1/IRE1/XBP1s pathway promotes the secretion of IL-6, IL-4, and PD-L1, thereby suppressing the functionality and proliferation of CAR-T cells and facilitating their apoptosis [147]. The mechanisms by which ERS affects tumor-associated DCs have also been elucidated, where changes in the TME leading to the accumulation of unfolded protein induce the activation of ERS and IRE1α–XBP1 signaling, thereby inhibiting their antigen-presenting function [148, 149]. These examples highlight the significant influence of the IRE1–XBP1 axis within ERS on tumor-related immune cells. Moreover, tumor-associated macrophages (TAMs), a predominant myeloid cell population in numerous cancers, exhibit increased XBP1 splicing in TAMs isolated from colorectal cancer patients compared to peripheral monocytes or macrophages [150]. The PERK pathway also influences the functionality of TAMs, as evidenced in the B16-F10 melanoma model, where PERK-deficient macrophages show impaired M2 polarization [151]. This illustrates the critical role of ERS in TAMs. The role of PERK in tumor immunity extends beyond this; a GSEA result indicates that PERK is primarily enriched in immune-related signaling pathways in breast cancer (BRCA), thyroid cancer, and head and neck squamous cell carcinoma (HNSSC). Furthermore, PERK expression correlates closely with infiltrating macrophages, DCs, and immune markers like macrophage mannose receptor 1 (MRC1, CD206), as well as T helper cells (Th) [152]. In the context of tumor-associated myeloid-derived suppressor cells (tumor-MDSCs), the downregulation of PERK transforms these cells into myeloid cells that activate CD8+ T cell-mediated anti-cancer immunity, featuring compromised NRF2-driven antioxidant capabilities and disrupted mitochondrial respiratory balance. This immune regulation is orchestrated through the STING pathway, with the abrogation of STING ameliorating the immune activation caused by PERK deficiency in tumor-MDSCs [153]. The fundamental mechanism involves STING’s ability to modulate NF-κB activity, a pivotal factor in the functionality of tumor-MDSCs [154].

In tumor response, the impact of ERS on tumor immunity also involves NK cells. As a downstream effector of the mTOR signaling pathway, IRE1–XBP1 regulates the proliferation of NK cells by targeting c-Myc [155]. Certainly, as a UPR effector protein, ATF6 plays a role in tumor-associated immune responses, and in the absence of inflammation, ATF6 activation in colon epithelial cells promotes intestinal dysbiosis and innate immune responses, leading to microbiota-dependent tumor formation, although the exact mechanism remains to be investigated [34]. In the UPR process, the immunoglobulin-binding protein BIP is crucial in tumor-associated immune dynamics. Downregulation of BIP through phosphorylation of PERK and IRE1 stimulates the generation of damage-associated molecular patterns in response to radiation-induced ERS, facilitating DC maturation and effector T lymphocyte activation. In addition, BIP gene knockout combined with irradiation of glioma stem cells effectively prevents tumor development and reduces tumor recurrence after radiotherapy [156].

## Discussion and prospects

The ER is a cellular component responsible for protein synthesis, and it induces ERS when its surrounding environment is disrupted or its function is impaired, disrupting the state of balance. ERS is widely associated with various human diseases, including neurodegenerative diseases, diabetes, cardiovascular diseases, inflammation, and cancers [19–21]. Pertinently, in cancer, ERS serves as a vital signal transduction pathway determining the fate of cancer cells, affecting their survival, metastasis, and resistance to therapy [157–159]. The TME is a process of polymorphic changes, such as hypoxia, glycation, and the accumulation of acidic substances, all of which can easily induce ERS [6, 7]. ERS broadly manifests through three principal pathways: the UPR, the endoplasmic overload response, and the sterol regulatory element-binding protein (SREBP) cascade. These mechanisms are instrumental in maintaining cellular homeostasis under stress conditions [8]. In tumor cells, the principal pathway involved is UPR, primarily orchestrated by IRE1, PERK, and ATF6. These three transmembrane proteins, located on the ER membrane, play distinct roles in manipulating tumor survival and development, alternately promoting or inhibiting tumor growth across different human tumors. Furthermore, the upregulation of ER-resident proteins and folding enzymes under stress conditions bolsters the ER’s protein-folding capacity, thereby alleviating the load of unfolded proteins within the ER lumen.

Numerous studies have shown that the UPR can promote the proliferation of tumors such as liver cancer, melanoma, and breast cancer through various signaling cascades such as IRE1/XBP1/JNK, IRE1/XBP1/IL-6/STAT3, and IRE1/TRAF2/NF-κB [23, 24, 28, 29]. In the context of ERS-mediated tumor regulation, the role of IRE1 emerges as particularly significant. This includes promoting the proliferation of breast cancer cells via its IRE1–XBP1 pathway [26]; mediating tumor cell apoptosis through the IRE1/TRAF2/ASK1/JNK pathway [47, 48], controlling IRE1 signaling via GPCRs and subsequently activating EMT to foster tumor metastasis [74]; or promoting tumor invasion through the IRE1–XBP1 pathway, reflecting the multifaceted role of IRE1 in tumor development. Similarly, PERK’s mechanism closely aligns with IRE1, though it may inhibit tumor proliferation, as evidenced in breast cancer [30]. ATF6 is mainly manifested in the acquisition of tumor resistance or sensitization, where it can promote tumor resistance via P58 [97], or act downstream of carfilzomib, achieving tumor resistance in patients resistant to cetuximab [99]. Additionally, ATF6’s role in promoting microbiome-dependent tumor formation through intestinal flora imbalance and innate immune responses [34], or facilitating prostate cancer progression via the ATF6α–PLA2G4A pathway affecting arachidonic acid metabolism [31], highlights its varied implications in cancer dynamics, albeit its limited reported involvement in angiogenesis, similar to PERK. Within the context of ERS, Bip/GRP78 functions as a critical chaperone protein, impacting tumor energy metabolism not only by directly modulating the expression of various metabolic regulators but also by displaying dual characteristics that augment both sensitization and resistance. While certain mechanisms have been elaborated upon, others remain enigmatic, such as whether Bip/GRP78 operates through the three downstream branches of the UPR or if additional cascading pathways are involved. Are there more exosomes that can regulate tumor cell resistance to immune agents? These questions continue to pose challenges. Throughout the various stages of tumor development influenced by ERS, it’s worth contemplating the cascading signals for tumor cell apoptosis elicited by ERS: (1) IRE1/TRAF2/ASK1/JNK pathway; (2) IRE1/TRAF2/caspase-12; (3) PERK/ATF4/CHOP; (4) IRE1–JNK–CHOP; (5) PERK/eIF2α/ATF4/CHOP/DR5; and (6) PERK/eIF2α/ATF4/CHOP/DR5 and other apoptotic pathways. These pathways predominantly achieve the induction of various tumor cell apoptosis by triggering the expression of caspase-3/7/9 or suppressing the expression of anti-apoptotic protein families. Hence, elucidating more about the mechanisms by which ERS incites tumor cell apoptosis is of paramount importance, with studies focusing on such pathways offering invaluable therapeutical targets. For instance, the P97 inhibitor CB-5083 activates the IRE1–XBP1s–CHOP pathway, promoting apoptosis in osteosarcoma cells [160]. In the treatment of neuroblastoma, resveratrol has been shown to trigger ERS-mediated intrinsic apoptosis in neuroblastoma cells and inhibit rho-dependent migration, thereby extending patient survival [161]. Recent studies have further demonstrated that fenretinide is an effective therapeutic option for neuroblastoma [162]. Beyond neuroblastoma, fenretinide also exhibits its anti-tumor effects by inducing apoptosis in head and neck squamous cell carcinoma through the upregulation of the pro-apoptotic protein NOXA via ERS [163]. These findings underscore the broad-spectrum anti-cancer properties of fenretinide. This highlights the potential of targeting ERS-induced apoptosis as an efficacious cancer treatment strategy.

Furthermore, ERS is pivotal in cancer cell survival, proliferation, and treatment resistance. Targeting ERS pathways, particularly the UPR mediated by IRE1, PERK, and ATF6, offers a novel therapeutic approach. As noted, IRE1 and PERK signaling are critical for RMS cell survival, with IRE1 inhibitor MKC8866 and PERK inhibitor AMGEN44 inducing senescence in rhabdomyosarcoma cells to curb proliferation. Therefore, developing RMS-targeted therapies using MKC8866 and AMGEN44 could be effective [164]. PDI family members are overexpressed in various cancers [165], and PDI dysfunction induces ERS, as previously discussed. For example, in PDAC cells, the PDI inhibitor E64FC26 induces ERS, disrupting protein homeostasis and leading to lysosomal defects. These defects limit apoptosis and ferroptosis induced by E64FC26-triggered ERS. Moreover, lysosomal defects prevent the formation of autophagolysosomes, resulting in autophagic cell death in PDAC cells [166]. Various PDI inhibitors, including LOC14 [167], securinine [168], and DDA [71], exhibit different effects across cancers, many closely linked to ERS, making them crucial targets for future development. ERS significantly contributes to tumor drug resistance. For instance, in EGFR(+) colon cancer patients resistant to cetuximab, carfilzomib can enhance apoptotic signaling through ERS, serving as a viable alternative to cetuximab [99]. Furthermore, the overexpression of Bip/GRP78 in cervical cancer cells results in cisplatin resistance, underscoring the therapeutic potential of targeting ERS in cancer treatment. Future research should aim to optimize these therapies and overcome potential resistance mechanisms [113]. In relation to the interplay between ERS and autophagy in tumor cells, this topic has been previously discussed. Clinically, chloroquine and its derivative hydroxychloroquine (HCQ) are the only autophagy inhibitors approved for use. Clinical studies have indicated that HCQ can overcome chemotherapy resistance in various tumor cell lines and animal cancer models [169]. Cook et al. showed that in a breast tumor rat model, Bip/GRP78 promotes acquired resistance to tamoxifen in tumor cells [170]. In ERα-positive breast cancer cell lines, combined treatment with HCQ and tamoxifen exhibited superior efficacy compared to endocrine monotherapy [171]. Other instances include apatinib inducing ERS-mediated apoptosis and autophagy in esophageal squamous cell carcinoma via the IRE1α–AKT–mTOR pathway, enhancing cell sensitivity to paclitaxel, and being used in combination with CQ [172]. These findings highlight the role of autophagy inhibitors in overcoming tumor resistance and sensitivity, suggesting that autophagy inhibitors represent a promising strategy for anti-cancer therapy.

Alterations in the tumor cell microenvironment and inherent stemness under ERS remain largely unexplored, necessitating urgent attention. Moreover, some crucial and complex issues persist unresolved, such as whether other chaperone molecules besides Bip might play a role in initiating the UPR pathway. A more comprehensive examination of the interactions and influences of the UPR’s tripartite branches IRE1, PERK, and ATF6 on tumor biological behaviors, including growth, invasion, metastasis, and angiogenesis, is imperative. Notably, these pathways often regulate tumor dynamics via downstream STAT3 signaling, underscoring the importance of exploring STAT3’s role in tumor pathology as a promising research direction. Additionally, the pressing need to develop new therapeutic targets countering ERS in cancer treatment cannot be overstated. Fortuitously, several strategies targeting ERS, such as GlaxoSmithKline’s PERK inhibitor GSK2656157 and the IRE1 activator IXA4/6, are already underway [173, 174]. Advancing the specificity of ERS’s regulatory influence on tumor biology remains a pivotal area for future exploration. Prospective studies should aim to systematically elucidate the role of ERS in tumor tissues, emphasize the effects of ERS-mediated signaling pathways on diverse tumor biological behaviors, and innovate targeted therapies based on ERS and its downstream mediators.

## References

[1] Jan CH, Williams CC, Weissman JS. Principles of ER cotranslational translocation revealed by proximity-specific ribosome profiling. Science. 2014;346:1257521. 10.1126/science.1257521. 25378630 10.1126/science.1257521 PMC4285348

[2] Reid DW, Nicchitta CV. Diversity and selectivity in mRNA translation on the endoplasmic reticulum. Nat Rev Mol Cell Biol. 2015;16:221–31. 10.1038/nrm3958. 25735911 10.1038/nrm3958 PMC4494666

[3] Wiseman RL, Mesgarzadeh JS, Hendershot LM. Reshaping endoplasmic reticulum quality control through the unfolded protein response. Mol Cell. 2022;82:1477–91. 10.1016/j.molcel.2022.03.025. 35452616 10.1016/j.molcel.2022.03.025 PMC9038009

[4] Vasic V, Denkert N, Schmidt CC, Riedel D, Stein A, Meinecke M. Hrd1 forms the retrotranslocation pore regulated by auto-ubiquitination and binding of misfolded proteins. Nat Cell Biol. 2020;22:274–81. 10.1038/s41556-020-0473-4. 32094691 10.1038/s41556-020-0473-4

[5] Christianson JC, Ye Y. Cleaning up in the endoplasmic reticulum: ubiquitin in charge. Nat Struct Mol Biol. 2014;21:325–35. 10.1038/nsmb.2793. 24699081 10.1038/nsmb.2793 PMC9397582

[6] Li A, Song NJ, Riesenberg BP, Li Z. The emerging roles of endoplasmic reticulum stress in balancing immunity and tolerance in health and diseases: mechanisms and opportunities. Front Immunol. 2019;10:3154. 10.3389/fimmu.2019.03154. 32117210 10.3389/fimmu.2019.03154 PMC7026265

[7] Luethy JD, Holbrook NJ. Activation of the gadd153 promoter by genotoxic agents: a rapid and specific response to DNA damage. Cancer Res. 1992;52:5–10. 1727386

[8] Cao T, Peng B, Zhou X, Cai J, Tang Y, Luo J, et al. Integrated signaling system under endoplasmic reticulum stress in eukaryotic microorganisms. Appl Microbiol Biotechnol. 2021;105:4805–18. 10.1007/s00253-021-11380-1. 34106312 10.1007/s00253-021-11380-1

[9] Hetz C, Zhang K, Kaufman RJ. Mechanisms, regulation and functions of the unfolded protein response. Nat Rev Mol Cell Biol. 2020;21:421–38. 10.1038/s41580-020-0250-z. 32457508 10.1038/s41580-020-0250-z PMC8867924

[10] Ron D, Hubbard SR. How IRE1 reacts to ER stress. Cell. 2008;132:24–26. 10.1016/j.cell.2007.12.017. 18191217 10.1016/j.cell.2007.12.017

[11] Kopp MC, Larburu N, Durairaj V, Adams CJ, Ali MMU. UPR proteins IRE1 and PERK switch BiP from chaperone to ER stress sensor. Nat Struct Mol Biol. 2019;26:1053–62. 10.1038/s41594-019-0324-9. 31695187 10.1038/s41594-019-0324-9 PMC6858872

[12] Almeida LM, Pinho BR, Duchen MR, Oliveira JMA. The PERKs of mitochondria protection during stress: insights for PERK modulation in neurodegenerative and metabolic diseases. Biol Rev Camb Philos Soc. 2022;97:1737–48. 10.1111/brv.12860. 35475315 10.1111/brv.12860

[13] Shen J, Prywes R. ER stress signaling by regulated proteolysis of ATF6. Methods. 2005;35:382–9. 10.1016/j.ymeth.2004.10.011. 15804611 10.1016/j.ymeth.2004.10.011

[14] Haze K, Yoshida H, Yanagi H, Yura T, Mori K. Mammalian transcription factor ATF6 is synthesized as a transmembrane protein and activated by proteolysis in response to endoplasmic reticulum stress. Mol Biol Cell. 1999;10:3787–99. 10.1091/mbc.10.11.3787. 10564271 10.1091/mbc.10.11.3787 PMC25679

[15] Yoshida H, Matsui T, Yamamoto A, Okada T, Mori K. XBP1 mRNA is induced by ATF6 and spliced by IRE1 in response to ER stress to produce a highly active transcription factor. Cell. 2001;107:881–91. 10.1016/s0092-8674(01)00611-0. 11779464 10.1016/s0092-8674(01)00611-0

[16] Yamamoto K, Sato T, Matsui T, Sato M, Okada T, Yoshida H, et al. Transcriptional induction of mammalian ER quality control proteins is mediated by single or combined action of ATF6alpha and XBP1. Dev Cell. 2007;13:365–76. 10.1016/j.devcel.2007.07.018. 17765680 10.1016/j.devcel.2007.07.018

[17] Wu J, Rutkowski DT, Dubois M, Swathirajan J, Saunders T, Wang J, et al. ATF6alpha optimizes long-term endoplasmic reticulum function to protect cells from chronic stress. Dev Cell. 2007;13:351–64. 10.1016/j.devcel.2007.07.005. 17765679 10.1016/j.devcel.2007.07.005

[18] Adachi Y, Yamamoto K, Okada T, Yoshida H, Harada A, Mori K. ATF6 is a transcription factor specializing in the regulation of quality control proteins in the endoplasmic reticulum. Cell Struct Funct. 2008;33:75–89. 10.1247/csf.07044. 18360008 10.1247/csf.07044

[19] Herrema H, Guan D, Choi JW, Feng X, Salazar Hernandez MA, Faruk F, et al. FKBP11 rewires UPR signaling to promote glucose homeostasis in type 2 diabetes and obesity. Cell Metab. 2022;34:1004–1022.e1008. 10.1016/j.cmet.2022.06.007. 35793654 10.1016/j.cmet.2022.06.007

[20] Poncet AF, Bosteels V, Hoffmann E, Chehade S, Rennen S, Huot L, et al. The UPR sensor IRE1alpha promotes dendritic cell responses to control *Toxoplasma gondii* infection. EMBO Rep. 2021;22:e49617. 10.15252/embr.201949617. 33586853 10.15252/embr.201949617 PMC7926260

[21] Wang L, Liu Y, Zhang X, Ye Y, Xiong X, Zhang S, et al. Endoplasmic reticulum stress and the unfolded protein response in cerebral ischemia/reperfusion injury. Front Cell Neurosci. 2022;16:864426. 10.3389/fncel.2022.864426. 35602556 10.3389/fncel.2022.864426 PMC9114642

[22] Hanahan D. Hallmarks of cancer: new dimensions. Cancer Discov. 2022;12:31–46. 10.1158/2159-8290.CD-21-1059. 35022204 10.1158/2159-8290.CD-21-1059

[23] Whyard T, Liu J, Darras FS, Waltzer WC, Romanov V. Organoid model of urothelial cancer: establishment and applications for bladder cancer research. Biotechniques. 2020;69:193–9. 10.2144/btn-2020-0068. 32654505 10.2144/btn-2020-0068

[24] Chen C, Zhang X. IRE1alpha-XBP1 pathway promotes melanoma progression by regulating IL-6/STAT3 signaling. J Transl Med. 2017;15:42. 10.1186/s12967-017-1147-2. 28222747 10.1186/s12967-017-1147-2 PMC5320675

[25] Chen J, Lei C, Zhang H, Huang X, Yang Y, Liu J, et al. RPL11 promotes non-small cell lung cancer cell proliferation by regulating endoplasmic reticulum stress and cell autophagy. BMC Mol Cell Biol. 2023;24:7 10.1186/s12860-023-00469-2. 36869281 10.1186/s12860-023-00469-2 PMC9985270

[26] Zhao N, Cao J, Xu L, Tang Q, Dobrolecki LE, Lv X, et al. Pharmacological targeting of MYC-regulated IRE1/XBP1 pathway suppresses MYC-driven breast cancer. J Clin Investig. 2018;128:1283–99. 10.1172/JCI95873. 29480818 10.1172/JCI95873 PMC5873887

[27] Wu CH, Silvers CR, Messing EM, Lee YF. Bladder cancer extracellular vesicles drive tumorigenesis by inducing the unfolded protein response in endoplasmic reticulum of nonmalignant cells. J Biol Chem. 2019;294:3207–18. 10.1074/jbc.RA118.006682. 30593508 10.1074/jbc.RA118.006682 PMC6398136

[28] Dolcet X, Llobet D, Pallares J, Matias-Guiu X. NF-kB in development and progression of human cancer. Virchows Arch. 2005;446:475–82. 10.1007/s00428-005-1264-9. 15856292 10.1007/s00428-005-1264-9

[29] Tam AB, Mercado EL, Hoffmann A, Niwa M. ER stress activates NF-kappaB by integrating functions of basal IKK activity, IRE1 and PERK. PLoS ONE. 2012;7:e45078 10.1371/journal.pone.0045078. 23110043 10.1371/journal.pone.0045078 PMC3482226

[30] Chitnis NS, Pytel D, Bobrovnikova-Marjon E, Pant D, Zheng H, Maas NL, et al. miR-211 is a prosurvival microRNA that regulates chop expression in a PERK-dependent manner. Mol Cell. 2012;48:353–64. 10.1016/j.molcel.2012.08.025. 23022383 10.1016/j.molcel.2012.08.025 PMC3496065

[31] Zhao R, Lv Y, Feng T, Zhang R, Ge L, Pan J, et al. ATF6α promotes prostate cancer progression by enhancing PLA2G4A-mediated arachidonic acid metabolism and protecting tumor cells against ferroptosis. Prostate. 2022;82:617–29. 10.1002/pros.24308. 35089606 10.1002/pros.24308 PMC9303695

[32] Feng T, Zhao R, Zhang H, Sun F, Hu J, Wang M, et al. Reciprocal negative feedback regulation of ATF6α and PTEN promotes prostate cancer progression. Cell Mol Life Sci. 2023;80:292. 10.1007/s00018-023-04940-3. 37715829 10.1007/s00018-023-04940-3 PMC11073217

[33] Benedetti R, Romeo MA, Arena A, Gilardini Montani MS, Di Renzo L, D’Orazi G, et al. ATF6 prevents DNA damage and cell death in colon cancer cells undergoing ER stress. Cell Death Discov. 2022;8:295. 10.1038/s41420-022-01085-3. 35752616 10.1038/s41420-022-01085-3 PMC9233702

[34] Coleman OI, Lobner EM, Bierwirth S, Sorbie A, Waldschmitt N, Rath E, et al. Activated ATF6 induces intestinal dysbiosis and innate immune response to promote colorectal tumorigenesis. Gastroenterology. 2018;155:1539–1552.e1512. 10.1053/j.gastro.2018.07.028. 30063920 10.1053/j.gastro.2018.07.028

[35] Liu F, Chang L, Hu J. Activating transcription factor 6 regulated cell growth, migration and inhibiteds cell apoptosis and autophagy via MAPK pathway in cervical cancer. J Reprod Immunol. 2020;139:103120. 10.1016/j.jri.2020.103120. 32234634 10.1016/j.jri.2020.103120

[36] Fan L, He Z, Head SA, Zhou Y, Lu T, Feng X, et al. Clofoctol and sorafenib inhibit prostate cancer growth via synergistic induction of endoplasmic reticulum stress and UPR pathways. Cancer Manag Res. 2018;10:4817–29. 10.2147/CMAR.S175256. 30425575 10.2147/CMAR.S175256 PMC6205540

[37] Zhao Y, Wang Y, Zhang L, Wang W, Fahey TJ, Yao K. BRAF inhibition promotes ER stress-mediated cell death in uveal melanoma. Neoplasma. 2022;69:1070–8. 10.4149/neo_2022_220428N462. 35786998 10.4149/neo_2022_220428N462

[38] Ma X, Li Y, Zhao B. Ribosomal protein L5 (RPL5)/ E2F transcription factor 1 (E2F1) signaling suppresses breast cancer progression via regulating endoplasmic reticulum stress and autophagy. Bioengineered. 2022;13:8076–86. 10.1080/21655979.2022.2052672. 35293275 10.1080/21655979.2022.2052672 PMC9161874

[39] Green DR, Levine B. To be or not to be? How selective autophagy and cell death govern cell fate. Cell. 2014;157:65–75. 10.1016/j.cell.2014.02.049. 24679527 10.1016/j.cell.2014.02.049 PMC4020175

[40] Song S, Tan J, Miao Y, Li M, Zhang Q. Crosstalk of autophagy and apoptosis: involvement of the dual role of autophagy under ER stress. J Cell Physiol. 2017;232:2977–84. 10.1002/jcp.25785. 28067409 10.1002/jcp.25785

[41] Deegan S, Saveljeva S, Gorman AM, Samali A. Stress-induced self-cannibalism: on the regulation of autophagy by endoplasmic reticulum stress. Cell Mol Life Sci. 2013;70:2425–41. 10.1007/s00018-012-1173-4. 23052213 10.1007/s00018-012-1173-4 PMC11113399

[42] Senft D, Ronai ZA. UPR, autophagy, and mitochondria crosstalk underlies the ER stress response. Trends Biochem Sci. 2015;40:141–8. 10.1016/j.tibs.2015.01.002. 25656104 10.1016/j.tibs.2015.01.002 PMC4340752

[43] Jia S, Xu X, Zhou S, Chen Y, Ding G, Cao L. Fisetin induces autophagy in pancreatic cancer cells via endoplasmic reticulum stress- and mitochondrial stress-dependent pathways. Cell Death Dis. 2019;10:142. 10.1038/s41419-019-1366-y. 30760707 10.1038/s41419-019-1366-y PMC6374379

[44] Wang J, Qi Q, Zhou W, Feng Z, Huang B, Chen A, et al. Inhibition of glioma growth by flavokawain B is mediated through endoplasmic reticulum stress induced autophagy. Autophagy. 2018;14:2007–22. 10.1080/15548627.2018.1501133. 30025493 10.1080/15548627.2018.1501133 PMC6152528

[45] Fang C, Weng T, Hu S, Yuan Z, Xiong H, Huang B, et al. IFN-γ-induced ER stress impairs autophagy and triggers apoptosis in lung cancer cells. Oncoimmunology. 2021;10:1962591. 10.1080/2162402X.2021.1962591. 34408924 10.1080/2162402X.2021.1962591 PMC8366549

[46] Li C, Zhang K, Pan G, Ji H, Li C, Wang X, et al. Dehydrodiisoeugenol inhibits colorectal cancer growth by endoplasmic reticulum stress-induced autophagic pathways. J Exp Clin Cancer Res. 2021;40:125. 10.1186/s13046-021-01915-9. 33838688 10.1186/s13046-021-01915-9 PMC8035743

[47] Urano F, Wang X, Bertolotti A, Zhang Y, Chung P, Harding HP, et al. Coupling of stress in the ER to activation of JNK protein kinases by transmembrane protein kinase IRE1. Science. 2000;287:664–6. 10.1126/science.287.5453.664. 10650002 10.1126/science.287.5453.664

[48] Nishitoh H, Matsuzawa A, Tobiume K, Saegusa K, Takeda K, Inoue K, et al. ASK1 is essential for endoplasmic reticulum stress-induced neuronal cell death triggered by expanded polyglutamine repeats. Genes Dev. 2002;16:1345–55. 10.1101/gad.992302. 12050113 10.1101/gad.992302 PMC186318

[49] Wu H, Zheng S, Zhang J, Xu S, Miao Z. Cadmium induces endoplasmic reticulum stress-mediated apoptosis in pig pancreas via the increase of Th1 cells. Toxicology. 2021;457:152790. 10.1016/j.tox.2021.152790. 33891997 10.1016/j.tox.2021.152790

[50] Lee H, Lee YS, Harenda Q, Pietrzak S, Oktay HZ, Schreiber S, et al. Beta cell dedifferentiation induced by IRE1α deletion prevents type 1 diabetes. Cell Metab. 2020;31:822–836.e825. 10.1016/j.cmet.2020.03.002. 32220307 10.1016/j.cmet.2020.03.002 PMC7346095

[51] Oyadomari S, Mori M. Roles of CHOP/GADD153 in endoplasmic reticulum stress. Cell Death Differ. 2004;11:381–9. 10.1038/sj.cdd.4401373. 14685163 10.1038/sj.cdd.4401373

[52] Hetz C. The unfolded protein response: controlling cell fate decisions under ER stress and beyond. Nat Rev Mol Cell Biol. 2012;13:89–102. 10.1038/nrm3270. 22251901 10.1038/nrm3270

[53] Kim TW, Lee SY, Kim M, Cheon C, Ko SG. Kaempferol induces autophagic cell death via IRE1-JNK-CHOP pathway and inhibition of G9a in gastric cancer cells. Cell Death Dis. 2018;9:875. 10.1038/s41419-018-0930-1. 30158521 10.1038/s41419-018-0930-1 PMC6115440

[54] Chen K, Jin P, He HH, Xie YH, Xie XY, Mo ZH. Overexpression of Insig-1 protects beta cell against glucolipotoxicity via SREBP-1c. J Biomed Sci. 2011;18:57. 10.1186/1423-0127-18-57. 21843373 10.1186/1423-0127-18-57 PMC3166905

[55] He C, Lu X, Li J, Shen K, Bai Y, Li Y, et al. The effect of quercetin on cervical cancer cells as determined by inducing tumor endoplasmic reticulum stress and apoptosis and its mechanism of action. Am J Transl Res. 2021;13:5240–7. 34150114 PMC8205781

[56] Peng KY, Chou TC. Osthole exerts inhibitory effects on hypoxic colon cancer cells via EIF2α phosphorylation-mediated apoptosis and regulation of HIFα. Am J Chin Med. 2022;50:621–37. 10.1142/S0192415X22500240. 35114913 10.1142/S0192415X22500240

[57] Wang TT, Yang Y, Wang F, Yang WG, Zhang JJ, Zou ZQ. Docosahexaenoic acid monoglyceride induces apoptosis and autophagy in breast cancer cells via lipid peroxidation-mediated endoplasmic reticulum stress. J Food Sci. 2021;86:4704–16. 10.1111/1750-3841.15900. 34494660 10.1111/1750-3841.15900

[58] Qi J, Zhou N, Li L, Mo S, Zhou Y, Deng Y, et al. Ciclopirox activates PERK-dependent endoplasmic reticulum stress to drive cell death in colorectal cancer. Cell Death Dis. 2020;11:582. 10.1038/s41419-020-02779-1. 32719342 10.1038/s41419-020-02779-1 PMC7385140

[59] Xu M, Zhu J, Liu S, Wang C, Shi Q, Kuang Y, et al. FOXD3, frequently methylated in colorectal cancer, acts as a tumor suppressor and induces tumor cell apoptosis under ER stress via p53. Carcinogenesis. 2020;41:1253–62. 10.1093/carcin/bgz198. 31784734 10.1093/carcin/bgz198

[60] Mora-Molina R, Stohr D, Rehm M, Lopez-Rivas A. cFLIP downregulation is an early event required for endoplasmic reticulum stress-induced apoptosis in tumor cells. Cell Death Dis. 2022;13:111. 10.1038/s41419-022-04574-6. 35115486 10.1038/s41419-022-04574-6 PMC8813907

[61] Wali VB, Bachawal SV, Sylvester PW. Endoplasmic reticulum stress mediates gamma-tocotrienol-induced apoptosis in mammary tumor cells. Apoptosis. 2009;14:1366–77. 10.1007/s10495-009-0406-y. 19771520 10.1007/s10495-009-0406-y

[62] Chang CY, Li JR, Wu CC, Wang JD, Liao SL, Chen WY, et al. Endoplasmic reticulum stress contributes to indomethacin-induced glioma apoptosis. Int J Mol Sci. 2020;21. 10.3390/ijms21020557. 10.3390/ijms21020557 PMC7013513 31952288

[63] Chang CY, Wu CC, Wang JD, Liao SL, Chen WY, Kuan YH, et al. Endoplasmic reticulum stress contributed to dipyridamole-induced impaired autophagic flux and glioma apoptosis. Int J Mol Sci. 2022;23. 10.3390/ijms23020579. 10.3390/ijms23020579 PMC8775759 35054765

[64] King AP, Marker SC, Swanda RV, Woods JJ, Qian SB, Wilson JJ. A rhenium isonitrile complex induces unfolded protein response-mediated apoptosis in cancer cells. Chemistry. 2019;25:9206–10. 10.1002/chem.201902223. 31090971 10.1002/chem.201902223 PMC6625872

[65] Kang N, Cao S, Jiang B, Zhang Q, Donkor PO, Zhu Y, et al. Cetuximab enhances oridonin-induced apoptosis through mitochondrial pathway and endoplasmic reticulum stress in laryngeal squamous cell carcinoma cells. Toxicol In Vitro. 2020;67:104885. 10.1016/j.tiv.2020.104885. 32407876 10.1016/j.tiv.2020.104885

[66] Lai WL, Wong NS. The PERK/eIF2α signaling pathway of unfolded protein response is essential for *N*-(4-hydroxyphenyl)retinamide (4HPR)-induced cytotoxicity in cancer cells. Exp Cell Res. 2008;314:1667–82. 10.1016/j.yexcr.2008.02.002. 18342855 10.1016/j.yexcr.2008.02.002

[67] Meares GP, Mines MA, Beurel E, Eom TY, Song L, Zmijewska AA, et al. Glycogen synthase kinase-3 regulates endoplasmic reticulum (ER) stress-induced CHOP expression in neuronal cells. Exp Cell Res. 2011;317:1621–8. 10.1016/j.yexcr.2011.02.012. 21356208 10.1016/j.yexcr.2011.02.012 PMC3103628

[68] Yang S, Jackson C, Karapetyan E, Dutta P, Kermah D, Wu Y, et al. Roles of protein disulfide isomerase in breast cancer. Cancers. 2022;14. 10.3390/cancers14030745. 10.3390/cancers14030745 PMC8833603 35159012

[69] Powell LE, Foster PA. Protein disulphide isomerase inhibition as a potential cancer therapeutic strategy. Cancer Med. 2021;10:2812–25. 10.1002/cam4.3836. 33742523 10.1002/cam4.3836 PMC8026947

[70] Wise R, Duhachek-Muggy S, Qi Y, Zolkiewski M, Zolkiewska A. Protein disulfide isomerases in the endoplasmic reticulum promote anchorage-independent growth of breast cancer cells. Breast Cancer Res Treat. 2016;157:241–52. 10.1007/s10549-016-3820-1. 27161215 10.1007/s10549-016-3820-1 PMC5662471

[71] Law ME, Yaaghubi E, Ghilardi AF, Davis BJ, Ferreira RB, Koh J, et al. Inhibitors of ERp44, PDIA1, and AGR2 induce disulfide-mediated oligomerization of death receptors 4 and 5 and cancer cell death. Cancer Lett. 2022;534:215604. 10.1016/j.canlet.2022.215604. 35247515 10.1016/j.canlet.2022.215604

[72] Shen T, Li Y, Chen Z, Liang S, Qiu Y, Zhu L, et al. Activating transcription factor 6 (ATF6) negatively regulates polo-like kinase 4 expression via recruiting C/EBPbeta to the upstream-promoter during ER stress. Biochim Biophys Acta Gene Regul Mech. 2020;1863:194488. 10.1016/j.bbagrm.2020.194488. 31926341 10.1016/j.bbagrm.2020.194488

[73] Lambert AW, Pattabiraman DR, Weinberg RA. Emerging biological principles of metastasis. Cell. 2017;168:670–91. 10.1016/j.cell.2016.11.037. 28187288 10.1016/j.cell.2016.11.037 PMC5308465

[74] Kumari N, Reabroi S, North BJ. Unraveling the molecular nexus between GPCRs, ERS, and EMT. Mediat Inflamm. 2021;2021:6655417. 10.1155/2021/6655417. 10.1155/2021/6655417 PMC7943314 33746610

[75] Sheng W, Wang G, Tang J, Shi X, Cao R, Sun J, et al. Calreticulin promotes EMT in pancreatic cancer via mediating Ca^2+^ dependent acute and chronic endoplasmic reticulum stress. J Exp Clin Cancer Res. 2020;39:209. 10.1186/s13046-020-01702-y. 33028359 10.1186/s13046-020-01702-y PMC7542892

[76] Gao L, Wang L, Dai T, Jin K, Zhang Z, Wang S, et al. Tumor-derived exosomes antagonize innate antiviral immunity. Nat Immunol. 2018;19:233–45. 10.1038/s41590-017-0043-5. 29358709 10.1038/s41590-017-0043-5

[77] Murakami T, Kawada K, Iwamoto M, Akagami M, Hida K, Nakanishi Y, et al. The role of CXCR3 and CXCR4 in colorectal cancer metastasis. Int J Cancer. 2013;132:276–87. 10.1002/ijc.27670. 22689289 10.1002/ijc.27670

[78] Lu C, Shi W, Hu W, Zhao Y, Zhao X, Dong F, et al. Endoplasmic reticulum stress promotes breast cancer cells to release exosomes circ_0001142 and induces M2 polarization of macrophages to regulate tumor progression. Pharmacol Res. 2022;177:106098. 10.1016/j.phrs.2022.106098. 35091089 10.1016/j.phrs.2022.106098

[79] Wang Z, Jiao P, Zhong Y, Ji H, Zhang Y, Song H, et al. The endoplasmic reticulum-stressed head and neck squamous cell carcinoma cells induced exosomal miR-424-5p inhibits angiogenesis and migration of humanumbilical vein endothelial cells through LAMC1-mediated Wnt/β-catenin signaling pathway. Cell Transplant. 2022;31:9636897221083549. 10.1177/09636897221083549. 35315295 10.1177/09636897221083549 PMC8943634

[80] Lin Y, Zhang C, Xiang P, Shen J, Sun W, Yu H. Exosomes derived from HeLa cells break down vascular integrity by triggering endoplasmic reticulum stress in endothelial cells. J Extracell Vesicles. 2020;9:1722385. 10.1080/20013078.2020.1722385. 32128072 10.1080/20013078.2020.1722385 PMC7034510

[81] Knutson AK, Williams AL, Boisvert WA, Shohet RV. HIF in the heart: development, metabolism, ischemia, and atherosclerosis. J Clin Investig. 2021;131. 10.1172/JCI137557. 10.1172/JCI137557 PMC8409592 34623330

[82] Romero-Ramirez L, Cao H, Regalado MP, Kambham N, Siemann D, Kim JJ, et al. X box-binding protein 1 regulates angiogenesis in human pancreatic adenocarcinomas. Transl Oncol. 2009;2:31–38. 10.1593/tlo.08211. 19252749 10.1593/tlo.08211 PMC2647700

[83] Moszynska A, Collawn JF, Bartoszewski R. IRE1 endoribonuclease activity modulates hypoxic HIF-1α signaling in human endothelial cells. Biomolecules. 2020;10. 10.3390/biom10060895. 10.3390/biom10060895 PMC7355874 32545307

[84] Auf G, Jabouille A, Guerit S, Pineau R, Delugin M, Bouchecareilh M, et al. Inositol-requiring enzyme 1α is a key regulator of angiogenesis and invasion in malignant glioma. Proc Natl Acad Sci USA. 2010;107:15553–8. 10.1073/pnas.0914072107. 20702765 10.1073/pnas.0914072107 PMC2932600

[85] Harnoss JM, Le Thomas A, Reichelt M, Guttman O, Wu TD, Marsters SA, et al. IRE1α disruption in triple-negative breast cancer cooperates with antiangiogenic therapy by reversing ER stress adaptation and remodeling the tumor microenvironment. Cancer Res. 2020;80:2368–79. 10.1158/0008-5472.CAN-19-3108. 32265225 10.1158/0008-5472.CAN-19-3108 PMC7272310

[86] Liu Y, Gray NS. Rational design of inhibitors that bind to inactive kinase conformations. Nat Chem Biol. 2006;2:358–64. 10.1038/nchembio799. 16783341 10.1038/nchembio799

[87] Carbajo-Lozoya J, Lutz S, Feng Y, Kroll J, Hammes HP, Wieland T. Angiotensin II modulates VEGF-driven angiogenesis by opposing effects of type 1 and type 2 receptor stimulation in the microvascular endothelium. Cell Signal. 2012;24:1261–9. 10.1016/j.cellsig.2012.02.005. 22374305 10.1016/j.cellsig.2012.02.005

[88] Fan F, Liu F, Shen P, Tao L, Zhang H, Wu H. Salvianolic acid B, a new type I IRE1 kinase inhibitor, abrogates AngII-induced angiogenesis by interacting with IRE1 in its active conformation. Clin Exp Pharmacol Physiol. 2023;50:82–95. 10.1111/1440-1681.13726. 36153795 10.1111/1440-1681.13726

[89] Sengupta S, Sharma CG, Jordan VC. Estrogen regulation of X-box binding protein-1 and its role in estrogen induced growth of breast and endometrial cancer cells. Horm Mol Biol Clin Investig. 2010;2:235–43. 10.1515/HMBCI.2010.025. 21297881 10.1515/HMBCI.2010.025 PMC3032413

[90] Pereira ER, Frudd K, Awad W, Hendershot LM. Endoplasmic reticulum (ER) stress and hypoxia response pathways interact to potentiate hypoxia-inducible factor 1 (HIF-1) transcriptional activity on targets like vascular endothelial growth factor (VEGF). J Biol Chem. 2014;289:3352–64. 10.1074/jbc.M113.507194. 24347168 10.1074/jbc.M113.507194 PMC3916539

[91] Ghosh R, Lipson KL, Sargent KE, Mercurio AM, Hunt JS, Ron D, et al. Transcriptional regulation of VEGF-A by the unfolded protein response pathway. PLoS ONE. 2010;5:e9575 10.1371/journal.pone.0009575. 20221394 10.1371/journal.pone.0009575 PMC2833197

[92] Wang Y, Alam GN, Ning Y, Visioli F, Dong Z, Nor JE, et al. The unfolded protein response induces the angiogenic switch in human tumor cells through the PERK/ATF4 pathway. Cancer Res. 2012;72:5396–406. 10.1158/0008-5472.CAN-12-0474. 22915762 10.1158/0008-5472.CAN-12-0474 PMC3743425

[93] Soni H, Bode J, Nguyen CDL, Puccio L, Nessling M, Piro RM, et al. PERK-mediated expression of peptidylglycine alpha-amidating monooxygenase supports angiogenesis in glioblastoma. Oncogenesis. 2020;9:18. 10.1038/s41389-020-0201-8. 32054826 10.1038/s41389-020-0201-8 PMC7018722

[94] Cai W, Sun X, Jin F, Xiao D, Li H, Sun H, et al. PERK-eIF2α-ERK1/2 axis drives mesenchymal-endothelial transition of cancer-associated fibroblasts in pancreatic cancer. Cancer Lett. 2021;515:86–95. 10.1016/j.canlet.2021.05.021. 34052329 10.1016/j.canlet.2021.05.021

[95] Bukowski K, Kciuk M, Kontek R. Mechanisms of multidrug resistance in cancer chemotherapy. Int J Mol Sci. 2020;21. 10.3390/ijms21093233. 10.3390/ijms21093233 PMC7247559 32370233

[96] Yu M, Lun J, Zhang H, Wang L, Zhang G, Zhang H, et al. Targeting UPR branches, a potential strategy for enhancing efficacy of cancer chemotherapy. Acta Biochim Biophys Sin. 2021;53:1417–27. 10.1093/abbs/gmab131. 34664059 10.1093/abbs/gmab131

[97] Khaled J, Kopsida M, Lennernas H, Heindryckx F. Drug resistance and endoplasmic reticulum stress in hepatocellular carcinoma. Cells. 2022;11. 10.3390/cells11040632. 10.3390/cells11040632 PMC8870354 35203283

[98] Khambata-Ford S, Garrett CR, Meropol NJ, Basik M, Harbison CT, Wu S, et al. Expression of epiregulin and amphiregulin and K-ras mutation status predict disease control in metastatic colorectal cancer patients treated with cetuximab. J Clin Oncol. 2007;25:3230–7. 10.1200/JCO.2006.10.5437. 17664471 10.1200/JCO.2006.10.5437

[99] Zulkifli A, Tan FH, Areeb Z, Stuart SF, Gomez J, Paradiso L, et al. Carfilzomib promotes the unfolded protein response and apoptosis in cetuximab-resistant colorectal cancer. Int J Mol Sci. 2021;22. 10.3390/ijms22137114. 10.3390/ijms22137114 PMC8269417 34281166

[100] Cirone M. Cancer cells dysregulate PI3K/AKT/mTOR pathway activation to ensure their survival and proliferation: mimicking them is a smart strategy of gammaherpesviruses. Crit Rev Biochem Mol Biol. 2021;56:500–9. 10.1080/10409238.2021.1934811. 34130564 10.1080/10409238.2021.1934811

[101] Salaroglio IC, Panada E, Moiso E, Buondonno I, Provero P, Rubinstein M, et al. PERK induces resistance to cell death elicited by endoplasmic reticulum stress and chemotherapy. Mol Cancer. 2017;16:91. 10.1186/s12943-017-0657-0. 28499449 10.1186/s12943-017-0657-0 PMC5427528

[102] Gu C, Zhang Y, Chen D, Liu H, Mi K. Tunicamycin-induced endoplasmic reticulum stress inhibits chemoresistance of FaDu hypopharyngeal carcinoma cells in 3D collagen I cultures and in vivo. Exp Cell Res. 2021;405:112725. 10.1016/j.yexcr.2021.112725. 34224701 10.1016/j.yexcr.2021.112725

[103] Gonzalez-Ortiz A, Pulido-Capiz A, Castaneda-Sanchez CY, Ibarra-Lopez E, Galindo-Hernandez O, Calderon-Fernandez MA, et al. eIF4A/PDCD4 pathway, a factor for doxorubicin chemoresistance in a triple-negative breast cancer cell model. Cells. 2022;11. 10.3390/cells11244069. 10.3390/cells11244069 PMC9776898 36552834

[104] Yan M, Ni J, Song D, Ding M, Huang J. Activation of unfolded protein response protects osteosarcoma cells from cisplatin-induced apoptosis through NF-kappaB pathway. Int J Clin Exp Pathol. 2015;8:10204–15. 26617729 PMC4637544

[105] Corazzari M, Rapino F, Ciccosanti F, Giglio P, Antonioli M, Conti B, et al. Oncogenic BRAF induces chronic ER stress condition resulting in increased basal autophagy and apoptotic resistance of cutaneous melanoma. Cell Death Differ. 2015;22:946–58. 10.1038/cdd.2014.183. 25361077 10.1038/cdd.2014.183 PMC4423179

[106] El-Khattouti A, Selimovic D, Hannig M, Taylor EB, Abd Elmageed ZY, Hassan SY, et al. Imiquimod-induced apoptosis of melanoma cells is mediated by ER stress-dependent Noxa induction and enhanced by NF-κB inhibition. J Cell Mol Med. 2016;20:266–86. 10.1111/jcmm.12718. 26578344 10.1111/jcmm.12718 PMC4727561

[107] Grzywa TM, Klicka K, Paskal W, Dudkiewicz J, Wejman J, Pyzlak M, et al. miR-410-3p is induced by vemurafenib via ER stress and contributes to resistance to BRAF inhibitor in melanoma. PLoS ONE. 2020;15:e0234707. 10.1371/journal.pone.0234707. 32555626 10.1371/journal.pone.0234707 PMC7299409

[108] Feng YH, Tung CL, Su YC, Tsao CJ, Wu TF. Proteomic profile of sorafenib resistance in hepatocellular carcinoma; GRP78 expression is associated with inferior response to sorafenib. Cancer Genom Proteom. 2019;16:569–76. 10.21873/cgp.20159. 10.21873/cgp.20159 PMC6885357 31659110

[109] Huang H, Gao Y, Liu A, Yang X, Huang F, Xu L, et al. EIF3D promotes sunitinib resistance of renal cell carcinoma by interacting with GRP78 and inhibiting its degradation. EBioMedicine. 2019;49:189–201. 10.1016/j.ebiom.2019.10.030. 31669222 10.1016/j.ebiom.2019.10.030 PMC6945244

[110] Liao CH, Tzeng YT, Lai GM, Chang CL, Hu MH, Tsai WL, et al. Omega-3 fatty acid-enriched fish oil and selenium combination modulates endoplasmic reticulum stress response elements and reverses acquired gefitinib resistance in HCC827 lung adenocarcinoma cells. Mar Drugs. 2020;18. 10.3390/md18080399. 10.3390/md18080399 PMC7460277 32751169

[111] Clarke WR, Amundadottir L, James MA. CLPTM1L/CRR9 ectodomain interaction with GRP78 at the cell surface signals for survival and chemoresistance upon ER stress in pancreatic adenocarcinoma cells. Int J Cancer. 2019;144:1367–78. 10.1002/ijc.32012. 30468251 10.1002/ijc.32012

[112] Xia T, Tian H, Zhang K, Zhang S, Chen W, Shi S, et al. Exosomal ERp44 derived from ER-stressed cells strengthens cisplatin resistance of nasopharyngeal carcinoma. BMC Cancer. 2021;21:1003. 10.1186/s12885-021-08712-9. 34493236 10.1186/s12885-021-08712-9 PMC8424889

[113] Luo C, Fan W, Jiang Y, Zhou S, Cheng W. Glucose-related protein 78 expression and its effects on cisplatin-resistance in cervical cancer. Med Sci Monit. 2018;24:2197–209. 10.12659/msm.906413. 29650944 10.12659/msm.906413 PMC5916091

[114] Li W, Wang W, Dong H, Li Y, Li L, Han L, et al. Cisplatin-induced senescence in ovarian cancer cells is mediated by GRP78. Oncol Rep. 2014;31:2525–34. 10.3892/or.2014.3147. 24756776 10.3892/or.2014.3147

[115] Zhou C, Li J, Du J, Jiang X, Xu X, Liu Y, et al. HMGCS1 drives drug-resistance in acute myeloid leukemia through endoplasmic reticulum-UPR-mitochondria axis. Biomed Pharmacother. 2021;137:111378. 10.1016/j.biopha.2021.111378. 33601148 10.1016/j.biopha.2021.111378

[116] Guo B, Xiong X, Hasani S, Wen YA, Li AT, Martinez R, et al. Downregulation of PHLPP induced by endoplasmic reticulum stress promotes eIF2α phosphorylation and chemoresistance in colon cancer. Cell Death Dis. 2021;12:960. 10.1038/s41419-021-04251-0. 34663797 10.1038/s41419-021-04251-0 PMC8523518

[117] Guo J, Zhong X, Tan Q, Yang S, Liao J, Zhuge J, et al. miR-301a-3p induced by endoplasmic reticulum stress mediates the occurrence and transmission of trastuzumab resistance in HER2-positive gastric cancer. Cell Death Dis. 2021;12:696. 10.1038/s41419-021-03991-3. 34257270 10.1038/s41419-021-03991-3 PMC8277821

[118] Kong M, Han Y, Zhao Y, Zhang H. miR-512-3p overcomes resistance to cisplatin in retinoblastoma by promoting apoptosis induced by endoplasmic reticulum stress. Med Sci Monit. 2020;26:e923817. 10.12659/MSM.923817. 32641679 10.12659/MSM.923817 PMC7370580

[119] Ricciardiello F, Gang Y, Palorini R, Li Q, Giampa M, Zhao F, et al. Hexosamine pathway inhibition overcomes pancreatic cancer resistance to gemcitabine through unfolded protein response and EGFR-Akt pathway modulation. Oncogene. 2020;39:4103–17. 10.1038/s41388-020-1260-1. 32235891 10.1038/s41388-020-1260-1

[120] Zhang XT, Hu XB, Wang HL, Kan WJ, Xu L, Wang ZJ, et al. Activation of unfolded protein response overcomes Ibrutinib resistance in diffuse large B-cell lymphoma. Acta Pharmacol Sin. 2021;42:814–23. 10.1038/s41401-020-00505-3. 32855532 10.1038/s41401-020-00505-3 PMC8115113

[121] Li Z, Ge Y, Dong J, Wang H, Zhao T, Wang X, et al. BZW1 facilitates glycolysis and promotes tumor growth in pancreatic ductal adenocarcinoma through potentiating eIF2α phosphorylation. Gastroenterology. 2022;162:1256–1271.e1214. 10.1053/j.gastro.2021.12.249. 34951995 10.1053/j.gastro.2021.12.249 PMC9436032

[122] Zhang J, Yang J, Lin C, Liu W, Huo Y, Yang M, et al. Endoplasmic reticulum stress-dependent expression of ERO1L promotes aerobic glycolysis in pancreatic cancer. Theranostics. 2020;10:8400–14. 10.7150/thno.45124. 32724477 10.7150/thno.45124 PMC7381747

[123] Poyyakkara A, Raji GR, Kunhiraman H, Edatt L, Kumar SVB. ER stress mediated regulation of miR23a confer Hela cells better adaptability to utilize glycolytic pathway. J Cell Biochem. 2018;119:4907–17. 10.1002/jcb.26718. 29377281 10.1002/jcb.26718

[124] Dauer P, Sharma NS, Gupta VK, Durden B, Hadad R, Banerjee S, et al. ER stress sensor, glucose regulatory protein 78 (GRP78) regulates redox status in pancreatic cancer thereby maintaining “stemness”. Cell Death Dis. 2019;10:132. 10.1038/s41419-019-1408-5. 30755605 10.1038/s41419-019-1408-5 PMC6372649

[125] Zheng Y, Liu P, Wang N, Wang S, Yang B, Li M, et al. Betulinic acid suppresses breast cancer metastasis by targeting GRP78-mediated glycolysis and ER stress apoptotic pathway. Oxid Med Cell Longev. 2019;2019:8781690. 10.1155/2019/8781690. 31531187 10.1155/2019/8781690 PMC6721262

[126] Wang Y, Chen S. TXNIP links anticipatory unfolded protein response to estrogen reprogramming glucose metabolism in breast cancer cells. Endocrinology. 2022;163. 10.1210/endocr/bqab212. 10.1210/endocr/bqab212 PMC8570585 34614512

[127] Wang C, Xu JL, Wen Y, Zhang DZ, Wang X, Chang L, et al. Fulvestrant inhibits the glycolysis of prolactinoma GH3 cells by downregulating IRE1/XBP1 signaling pathway. Eur Rev Med Pharmacol Sci. 2018;22:5364–70. 10.26355/eurrev_201808_15739. 30178863 10.26355/eurrev_201808_15739

[128] Fouad YA, Aanei C. Revisiting the hallmarks of cancer. Am J Cancer Res. 2017;7:1016–36. 28560055 PMC5446472

[129] Xie H, Tang CH, Song JH, Mancuso A, Del Valle JR, Cao J, et al. IRE1α RNase-dependent lipid homeostasis promotes survival in Myc-transformed cancers. J Clin Investig. 2018;128:1300–16. 10.1172/JCI95864. 29381485 10.1172/JCI95864 PMC5873889

[130] Wang H, Zhang Y, Lu Y, Song J, Huang M, Zhang J, et al. The role of stearoyl-coenzyme A desaturase 1 in clear cell renal cell carcinoma. Tumour Biol. 2016;37:479–89. 10.1007/s13277-015-3451-x. 26224474 10.1007/s13277-015-3451-x

[131] Almanza A, Mnich K, Blomme A, Robinson CM, Rodriguez-Blanco G, Kierszniowska S, et al. Regulated IRE1α-dependent decay (RIDD)-mediated reprograming of lipid metabolism in cancer. Nat Commun. 2022;13:2493. 10.1038/s41467-022-30159-0. 35524156 10.1038/s41467-022-30159-0 PMC9076827

[132] Bobrovnikova-Marjon E, Hatzivassiliou G, Grigoriadou C, Romero M, Cavener DR, Thompson CB, et al. PERK-dependent regulation of lipogenesis during mouse mammary gland development and adipocyte differentiation. Proc Natl Acad Sci USA. 2008;105:16314–9. 10.1073/pnas.0808517105. 18852460 10.1073/pnas.0808517105 PMC2570995

[133] Zhu Y, Li Y, Bai B, Shang C, Fang J, Cong J, et al. Effects of apoptin-induced endoplasmic reticulum stress on lipid metabolism, migration, and invasion of HepG-2 cells. Front Oncol. 2021;11:614082 10.3389/fonc.2021.614082. 33718168 10.3389/fonc.2021.614082 PMC7952871

[134] Chen X, Cubillos-Ruiz JR. Endoplasmic reticulum stress signals in the tumour and its microenvironment. Nat Rev Cancer. 2021;21:71–88. 10.1038/s41568-020-00312-2. 33214692 10.1038/s41568-020-00312-2 PMC7927882

[135] Thommen DS, Schumacher TN. T cell dysfunction in cancer. Cancer Cell. 2018;33:547–62. 10.1016/j.ccell.2018.03.012. 29634943 10.1016/j.ccell.2018.03.012 PMC7116508

[136] Blank CU, Haining WN, Held W, Hogan PG, Kallies A, Lugli E, et al. Defining ‘T cell exhaustion’. Nat Rev Immunol. 2019;19:665–74. 10.1038/s41577-019-0221-9. 31570879 10.1038/s41577-019-0221-9 PMC7286441

[137] Hurst KE, Lawrence KA, Essman MT, Walton ZJ, Leddy LR, Thaxton JE. Endoplasmic reticulum stress contributes to mitochondrial exhaustion of CD8^+^ T cells. Cancer Immunol Res. 2019;7:476–86. 10.1158/2326-6066.CIR-18-0182. 30659052 10.1158/2326-6066.CIR-18-0182 PMC6397687

[138] Cao Y, Trillo-Tinoco J, Sierra RA, Anadon C, Dai W, Mohamed E, et al. ER stress-induced mediator C/EBP homologous protein thwarts effector T cell activity in tumors through T-bet repression. Nat Commun. 2019;10:1280 10.1038/s41467-019-09263-1. 30894532 10.1038/s41467-019-09263-1 PMC6426975

[139] Song M, Sandoval TA, Chae CS, Chopra S, Tan C, Rutkowski MR, et al. IRE1α-XBP1 controls T cell function in ovarian cancer by regulating mitochondrial activity. Nature. 2018;562:423–8. 10.1038/s41586-018-0597-x. 30305738 10.1038/s41586-018-0597-x PMC6237282

[140] Ma X, Bi E, Lu Y, Su P, Huang C, Liu L, et al. Cholesterol induces CD8^+^ T cell exhaustion in the tumor microenvironment. Cell Metab. 2019;30:143–156 e145. 10.1016/j.cmet.2019.04.002. 31031094 10.1016/j.cmet.2019.04.002 PMC7061417

[141] Ma X, Xiao L, Liu L, Ye L, Su P, Bi E, et al. CD36-mediated ferroptosis dampens intratumoral CD8^+^ T cell effector function and impairs their antitumor ability. Cell Metab. 2021;33:1001–1012.e1005. 10.1016/j.cmet.2021.02.015. 33691090 10.1016/j.cmet.2021.02.015 PMC8102368

[142] Xu S, Chaudhary O, Rodriguez-Morales P, Sun X, Chen D, Zappasodi R, et al. Uptake of oxidized lipids by the scavenger receptor CD36 promotes lipid peroxidation and dysfunction in CD8^+^ T cells in tumors. Immunity. 2021;54:1561–1577.e1567. 10.1016/j.immuni.2021.05.003. 34102100 10.1016/j.immuni.2021.05.003 PMC9273026

[143] Zhang X, Qiu J, Huang F, Han P, Shan K, Zhang C. Construction and verification of a hypoxia-related nine-gene prognostic model in uveal melanoma based on integrated single-cell and bulk RNA sequencing analyses. Exp Eye Res. 2022;223:109214. 10.1016/j.exer.2022.109214. 35981602 10.1016/j.exer.2022.109214

[144] Mahadevan NR, Anufreichik V, Rodvold JJ, Chiu KT, Sepulveda H, Zanetti M. Cell-extrinsic effects of tumor ER stress imprint myeloid dendritic cells and impair CD8^+^ T cell priming. PLoS ONE. 2012;7:e51845. 10.1371/journal.pone.0051845. 23272178 10.1371/journal.pone.0051845 PMC3525659

[145] Zhu C, Dixon KO, Newcomer K, Gu G, Xiao S, Zaghouani S, et al. Tim-3 adaptor protein Bat3 is a molecular checkpoint of T cell terminal differentiation and exhaustion. Sci Adv. 2021;7. 10.1126/sciadv.abd2710. 10.1126/sciadv.abd2710 PMC8087420 33931442

[146] Cubillos-Ruiz JR, Silberman PC, Rutkowski MR, Chopra S, Perales-Puchalt A, Song M, et al. ER stress sensor XBP1 controls anti-tumor immunity by disrupting dendritic cell homeostasis. Cell. 2015;161:1527–38. 10.1016/j.cell.2015.05.025. 26073941 10.1016/j.cell.2015.05.025 PMC4580135

[147] Li W, Wu L, Huang C, Ma H, Wang L, Liu W, et al. Activation of Notch-1 signaling pathway in macrophages to secrete PD-L1 and regulate cytotoxicity of CAR-T cells in diffuse large B-cell lymphoma. Aging. 2024;16:1845–59. 10.18632/aging.205463. 38261741 10.18632/aging.205463 PMC10866421

[148] Cubillos-Ruiz JR, Bettigole SE, Glimcher LH. Tumorigenic and immunosuppressive effects of endoplasmic reticulum stress in cancer. Cell. 2017;168:692–706. 10.1016/j.cell.2016.12.004. 28187289 10.1016/j.cell.2016.12.004 PMC5333759

[149] Giovanelli P, Sandoval TA, Cubillos-Ruiz JR. Dendritic cell metabolism and function in tumors. Trends Immunol. 2019;40:699–718. 10.1016/j.it.2019.06.004. 31301952 10.1016/j.it.2019.06.004

[150] Zhao Y, Zhang W, Huo M, Wang P, Liu X, Wang Y, et al. XBP1 regulates the protumoral function of tumor-associated macrophages in human colorectal cancer. Signal Transduct Target Ther. 2021;6:357. 10.1038/s41392-021-00761-7. 34667145 10.1038/s41392-021-00761-7 PMC8526672

[151] Raines LN, Zhao H, Wang Y, Chen HY, Gallart-Ayala H, Hsueh PC, et al. PERK is a critical metabolic hub for immunosuppressive function in macrophages. Nat Immunol. 2022;23:431–45. 10.1038/s41590-022-01145-x. 35228694 10.1038/s41590-022-01145-x PMC9112288

[152] Tang L, Xiong W, Zhang L, Wang D, Wang Y, Wu Y, et al. circSETD3 regulates MAPRE1 through miR-615-5p and miR-1538 sponges to promote migration and invasion in nasopharyngeal carcinoma. Oncogene. 2021;40:307–21. 10.1038/s41388-020-01531-5. 33122825 10.1038/s41388-020-01531-5

[153] Mohamed E, Sierra RA, Trillo-Tinoco J, Cao Y, Innamarato P, Payne KK, et al. The unfolded protein response mediator PERK governs myeloid cell-driven immunosuppression in tumors through inhibition of STING signaling. Immunity. 2020;52:668–682.e667. 10.1016/j.immuni.2020.03.004. 32294407 10.1016/j.immuni.2020.03.004 PMC7207019

[154] Sierra RA, Trillo-Tinoco J, Mohamed E, Yu L, Achyut BR, Arbab A, et al. Anti-jagged immunotherapy inhibits MDSCs and overcomes tumor-induced tolerance. Cancer Res. 2017;77:5628–38. 10.1158/0008-5472.CAN-17-0357. 28904063 10.1158/0008-5472.CAN-17-0357 PMC5679354

[155] Dong H, Adams NM, Xu Y, Cao J, Allan DSJ, Carlyle JR, et al. The IRE1 endoplasmic reticulum stress sensor activates natural killer cell immunity in part by regulating c-Myc. Nat Immunol. 2019;20:865–78. 10.1038/s41590-019-0388-z. 31086333 10.1038/s41590-019-0388-z PMC6588410

[156] Yang W, Xiu Z, He Y, Huang W, Li Y, Sun T. Bip inhibition in glioma stem cells promotes radiation-induced immunogenic cell death. Cell Death Dis. 2020;11:786. 10.1038/s41419-020-03000-z. 32963254 10.1038/s41419-020-03000-z PMC7508950

[157] van Ziel AM, Scheper W. The UPR in neurodegenerative disease: not just an inside job. Biomolecules. 2020;10. 10.3390/biom10081090. 10.3390/biom10081090 PMC7465596 32707908

[158] Kang Z, Chen F, Wu W, Liu R, Chen T, Xu F. UPR(mt) and coordinated UPR(ER) in type 2 diabetes. Front Cell Dev Biol. 2022;10:974083. 10.3389/fcell.2022.974083. 36187475 10.3389/fcell.2022.974083 PMC9523447

[159] Ren J, Bi Y, Sowers JR, Hetz C, Zhang Y. Endoplasmic reticulum stress and unfolded protein response in cardiovascular diseases. Nat Rev Cardiol. 2021;18:499–521. 10.1038/s41569-021-00511-w. 33619348 10.1038/s41569-021-00511-w

[160] Zhao Z, Wu M, Zhang X, Jin Q, Wang Y, Zou C, et al. CB-5083, an inhibitor of P97, suppresses osteosarcoma growth and stem cell properties by altering protein homeostasis. Am J Transl Res. 2020;12:2956–67. 32655822 PMC7344079

[161] Sun DP, Chen JT, Yang ST, Chen TH, Liu SH, Chen RM. Resveratrol triggers the ER stress-mediated intrinsic apoptosis of neuroblastoma cells coupled with suppression of Rho-dependent migration and consequently prolongs mouse survival. Chem Biol Interact. 2023;382:110645. 10.1016/j.cbi.2023.110645. 37482209 10.1016/j.cbi.2023.110645

[162] Bensa V, Calarco E, Giusto E, Perri P, Corrias MV, Ponzoni M, et al. Retinoids delivery systems in cancer: liposomal fenretinide for neuroectodermal-derived tumors. Pharmaceuticals*.* 2021;14. 10.3390/ph14090854. 10.3390/ph14090854 PMC8466194 34577553

[163] Britt EL, Raman S, Leek K, Sheehy CH, Kim SW, Harada H. Combination of fenretinide and ABT-263 induces apoptosis through NOXA for head and neck squamous cell carcinoma treatment. PLoS ONE. 2019;14:e0219398. 10.1371/journal.pone.0219398. 31276572 10.1371/journal.pone.0219398 PMC6611623

[164] McCarthy N, Dolgikh N, Logue S, Patterson JB, Zeng Q, Gorman AM, et al. The IRE1 and PERK arms of the unfolded protein response promote survival of rhabdomyosarcoma cells. Cancer Lett. 2020;490:76–88. 10.1016/j.canlet.2020.07.009. 32679165 10.1016/j.canlet.2020.07.009

[165] Hoffstrom BG, Kaplan A, Letso R, Schmid RS, Turmel GJ, Lo DC, et al. Inhibitors of protein disulfide isomerase suppress apoptosis induced by misfolded proteins. Nat Chem Biol. 2010;6:900–6. 10.1038/nchembio.467. 21079601 10.1038/nchembio.467 PMC3018711

[166] Hung CS, Lee KL, Huang WJ, Su FH, Liang YC. Pan-inhibition of protein disulfide isomerase caused cell death through disrupting cellular proteostasis in pancreatic ductal adenocarcinoma cells. Int J Mol Sci. 2023;24. 10.3390/ijms242216467. 10.3390/ijms242216467 PMC10671009 38003657

[167] Kaplan A, Gaschler MM, Dunn DE, Colligan R, Brown LM, Palmer AG, et al. Small molecule-induced oxidation of protein disulfide isomerase is neuroprotective. Proc Natl Acad Sci USA. 2015;112:E2245–2252. 10.1073/pnas.1500439112. 25848045 10.1073/pnas.1500439112 PMC4418888

[168] Kaplan A, Stockwell BR. Structural elucidation of a small molecule inhibitor of protein disulfide isomerase. ACS Med Chem Lett. 2015;6:966–71. 10.1021/acsmedchemlett.5b00014. 26500720 10.1021/acsmedchemlett.5b00014 PMC4603724

[169] Cook KL, Warri A, Soto-Pantoja DR, Clarke PA, Cruz MI, Zwart A, et al. Hydroxychloroquine inhibits autophagy to potentiate antiestrogen responsiveness in ER+ breast cancer. Clin Cancer Res. 2014;20:3222–32. 10.1158/1078-0432.CCR-13-3227. 24928945 10.1158/1078-0432.CCR-13-3227 PMC4073207

[170] Cook KL, Shajahan AN, Warri A, Jin L, Hilakivi-Clarke LA, Clarke R. Glucose-regulated protein 78 controls cross-talk between apoptosis and autophagy to determine antiestrogen responsiveness. Cancer Res. 2012;72:3337–49. 10.1158/0008-5472.CAN-12-0269. 22752300 10.1158/0008-5472.CAN-12-0269 PMC3576872

[171] Chude CI, Amaravadi RK. Targeting autophagy in cancer: update on clinical trials and novel inhibitors. Int J Mol Sci. 2017;18. 10.3390/ijms18061279. 10.3390/ijms18061279 PMC5486101 28621712

[172] Wang YM, Xu X, Tang J, Sun ZY, Fu YJ, Zhao XJ, et al. Apatinib induces endoplasmic reticulum stress-mediated apoptosis and autophagy and potentiates cell sensitivity to paclitaxel via the IRE-1α-AKT-mTOR pathway in esophageal squamous cell carcinoma. Cell Biosci. 2021;11:124. 10.1186/s13578-021-00640-2. 34229754 10.1186/s13578-021-00640-2 PMC8261945

[173] Rojas-Rivera D, Delvaeye T, Roelandt R, Nerinckx W, Augustyns K, Vandenabeele P, et al. When PERK inhibitors turn out to be new potent RIPK1 inhibitors: critical issues on the specificity and use of GSK2606414 and GSK2656157. Cell Death Differ. 2017;24:1100–10. 10.1038/cdd.2017.58. 28452996 10.1038/cdd.2017.58 PMC5442476

[174] Grandjean JMD, Madhavan A, Cech L, Seguinot BO, Paxman RJ, Smith E, et al. Pharmacologic IRE1/XBP1s activation confers targeted ER proteostasis reprogramming. Nat Chem Biol. 2020;16:1052–61. 10.1038/s41589-020-0584-z. 32690944 10.1038/s41589-020-0584-z PMC7502540

